# Adaptive evolution characteristics of mitochondrial genomes in genus *Aparapotamon* (Brachyura, Potamidae) of freshwater crabs

**DOI:** 10.1186/s12864-023-09290-9

**Published:** 2023-04-12

**Authors:** Yu-Tong Ji, Xiao-Juan Zhou, Qian Yang, Yuan-Biao Lu, Jun Wang, Jie-Xin Zou

**Affiliations:** 1 Research Laboratory of Freshwater Crustacean Decapoda & Paragonimus, School of Basic Medical Sciences, Nanchang University, 461 Bayi Avenue, Nanchang City, 330006 Jiangxi Province China; 2 Department of Parasitology, School of Basic Medical Science, Nanchang University, 461 Bayi Avenue, Nanchang City, 330006 Jiangxi Province China

**Keywords:** Freshwater crab, Genus *Aparapotamon*, Comparative genomics, Mitochondrial genome, Phylogeny, Adaptive evolution

## Abstract

**Background:**

*Aparapotamon*, a freshwater crab genus endemic to China, includes 13 species. The distribution of *Aparapotamon* spans the first and second tiers of China's terrain ladder, showing great altitudinal differences. To study the molecular mechanisms of adaptive evolution in *Aparapotamon*, we performed evolutionary analyses, including morphological, geographical, and phylogenetic analyses and divergence time estimation. We sequenced the mitogenomes of *Aparapotamon binchuanense* and *Aparapotamon huizeense* for the first time and resequenced three other mitogenomes of *Aparapotamon grahami* and *Aparapotamon gracilipedum*. These sequences were combined with NCBI sequences to perform comparative mitogenome analysis of all 13 *Aparapotamon* species, revealing mitogenome arrangement and the characteristics of protein-coding and tRNA genes.

**Results:**

A new species classification scheme of the genus *Aparapotamon* has been detected and verified by different aspects, including geographical, morphological, phylogenetics and comparative mitogenome analyses. Imprints from adaptive evolution were discovered in the mitochondrial genomes of group A, including the same codon loss at position 416 of the *ND6* gene and the unique arrangement pattern of the *tRNA-Ile* gene*.* Multiple tRNA genes conserved or involved in adaptive evolution were detected. Two genes associated with altitudinal adaptation, *ATP8* and *ND6,* which experienced positive selection, were identified for the first time in freshwater crabs.

**Conclusions:**

Geological movements of the Qinghai-Tibet Plateau and Hengduan Mountains likely strongly impacted the speciation and differentiation of the four *Aparapotamon* groups. After some group A species dispersed from the Hengduan Mountain Range, new evolutionary characteristics emerged in their mitochondrial genomes, facilitating adaptation to the low-altitude environment of China's second terrain tier. Ultimately, group A species spread to high latitudes along the upper reaches of the Yangtze River, showing faster evolutionary rates, higher species diversity and the widest distribution.

**Supplementary Information:**

The online version contains supplementary material available at 10.1186/s12864-023-09290-9.

## Background

Potamidae (Ortmann, 1896), a family of freshwater crabs belonging to Brachyura, is mainly distributed in Asia, Europe and Africa [1]. With more than 318 species in 52 genera reported to date, China has the richest species diversity of Potamidae worldwide [2, 3]. Due to the constantly studied and updated taxonomy and the discovery of new freshwater crab species, the number is still rising [4–6]. The freshwater crab species endemic to China show great diversity in terms of genetic, morphological and geographical distribution patterns [7]. However, as a result of climate changes and human-driven destruction of ecological habitats, the survival of freshwater crabs has been affected in recent years [8, 9]. Related research and conservation are urgently needed. Meanwhile, studies have shown that in China, more than 100 species of freshwater crabs can serve as secondary intermediate hosts for *Paragonimus* [10]. Therefore, by further understanding the taxonomy and evolutionary history of Potamidae, the potential coevolutionary relationship between the two can also be preliminarily discussed.

Freshwater crabs are amphibious species that spend their lives both on land and in water and can complete their life cycle outside an ocean environment [11]. Unlike most Brachyura, which undergo a free-living larval stage, freshwater crabs can directly hatch from yolky eggs into mature crabs [12], a feature that is relatively stable and shows obvious geographical isolation. Therefore, the distribution of different freshwater crabs is easily influenced by factors such as mountainous terrain and climate fluctuations due to their reproductive characteristics and weak migration behaviours [7, 13, 14]. All these features make freshwater crabs a very suitable model for phylogenetic analysis [15, 16], as well as for studying the influence of historical paleogeological events on speciation [17, 18].

*Aparapotamon* (Dai et Chen, 1985) is an endemic freshwater genus distributed in China that includes 13 species reported to date [3, 6]. As early as 1929, *Aparapotamon grahami* (Rathbun, 1929) was first reported and then discovered in various locations, including six provinces (Yunnan, Sichuan, Guizhou, Hunan, Hubei and Henan) in China [3, 7]. From 1980 to 1990, ten *Aparapotamon* species were discovered and reported, namely, *Aparapotamon gracilipedum* (Chen et Chang, 1982) and another nine *Aparapotamon* species (Dai et Chen, 1985). Thereafter, there were no reports of any new *Aparapotamon* species until 2021, when our research laboratory reported two new species, *Aparapotamon binchuanense* (Tan et al., 2021) and *Aparapotamon huizeense* (Tan et al., 2021), increasing the diversity of the genus [6].

The geographical distribution range of the genus *Aparapotamon* crosses geographical barriers created by multiple mountains and river systems in China [3]. Meanwhile, we found that the Hengduan Mountain Range is the primary geographical distribution area of the genus *Aparapotamon*. On the southeastern Qinghai-Tibet Plateau, the Hengduan Mountain Range is one of the world's most important biodiversity hotspots with active geological movements [19–21] and is the demarcation line of the first and second tiers of China's terrain, dividing the Qinghai–Tibet Plateau and Yunnan-Kweichow Plateau. The Hengduan Mountain Range was also an important shelter and centre of species origin during the glacial period, with rich species diversity and unique geological characteristics [22, 23]. The adaptive evolutionary mechanisms of species in this region have attracted much attention [24, 25]. However, the current taxonomy and evolutionary mechanisms within the genus *Aparapotamon* are not very clear and detailed. Therefore, based on all related specimens in our library and other published studies on the genus *Aparapotamon*, morphological classification and geographical distribution analysis were carried out on all 13 *Aparapotamon* species, aiming to explore the evolutionary relationships within the genus *Aparapotamon*.

As the carrier of extranuclear genetic material, mitochondrial genomes present specificities such as maternal inheritance and a rapid evolutionary rate [26]. Therefore, they have been widely used in various studies, such as those on genetic diversity [27, 28], phylogenetic analysis [29, 30] and biogeography [31]. The mitochondrial gene cytochrome oxidase subunit I (*COX1*) has been commonly used as a molecular marker for phylogenetic analysis of freshwater crabs and is considered a DNA barcode [18, 32, 33]. Other nuclear genes, such as *28S rRNA,* are also used as supplements to increase the credibility of the results [34]. Due to the limitations of studies based on single-gene molecular markers and the accessibility of the complete mitochondrial genome [35], comparative mitogenome analysis has widely been carried out in studies on species diversification [33, 36, 37]. In this study, five complete mitogenomes were sequenced, and the mitogenome sequences of all 13 species in the genus *Aparapotamon* were analysed, revealing the molecular mechanism of species diversification.

Combining all the findings together, including those of morphology, geography, phylogenetic relationships and mitogenome comparisons, we were surprised to find much evidence for the existence of different groups within the genus *Aparapotamon*. Therefore, a new classification scheme within the genus *Aparapotamon* was proposed, which divided all 13 *Aparapotamon* species into four groups. This conclusion is largely supported by the results of this study, representing a considerable step forward in the exploration of relationships within the genus *Aparapotamon*. Meanwhile, in this study, species diversification and adaptive evolution patterns were also discussed at the genomic level. Combining the morphology and geographical distribution results, ancestral reconstruction within the genus *Aparapotamon* can finally be achieved. The results will help reveal the molecular mechanisms underlying the species diversity of freshwater crabs in China as well as contribute to species diversity conservation in China.

## Materials and methods

### Sample collection and specimens

In this study, 357 *Aparapotamon* individual from seven species were collected from 21 collection locations in five provinces in China, including Yunnan, Guizhou, Sichuan, Chongqing and Henan (See Supplementary Table 1, Additional file 1). After collection, the specimens were fixed immediately in 95% ethanol at 4 °C. All the specimens were preserved in the Freshwater Crustacean Decapoda Research Laboratory of School of Basic Medical Sciences, Nanchang University. The present distribution localities of all 13 species in genus *Aparapotamon* were mapped using Arcgis10.2 software based on the known records and the sampling sites in this study (See Supplementary Table 2, Additional file 1). The map dataset was provided by the Geospatial Data Cloud [38].

### Morphological examination

According to the morphological descriptions in Fauna Sinica (1999) [3] and the holotype database of freshwater crabs established by our laboratory, the morphological identification and classification of the specimens in the *Aparapotamon* genus were performed. The morphological characteristics were observed by stereo microscope. Digital Caliper was used to measure the morphological features, such as the length and width of the carapace. The important morphological regions, which mainly include carapace, chelipeds, male first gonopod (G1) and ambulatory legs, were photographed with a Nikon D500 Digital Camera.

The morphology of G1 is most meaningful in freshwater crab species identification among all morphological factors [39]. The slight differences between G1 in the samples can be observed. Through the geographical distributions and G1 morphological features, the identification and classification of the *Aparapotamon* genus were verified. Since the morphological differences between different species are tiny, only morphological observation is not valid in species identification, which proves the significance of further molecular analysis.

### DNA extraction, PCR and sequencing

In this study, DNA extraction was carried out by E.Z.N.A.® Mollusc DNA kit (D3373) according to the DNA kit instructions. The chloroform and isoamyl alcohol in a 24:1 volume ratio was prepared to extract the nucleic acid. The muscular tissue from ambulatory legs was sampled in the microtubes for homogenization and DNA extraction. The total DNA was extracted and stored at -20 °C.

The target mitochondrial gene *COX1*, *16S rRNA* and nuclear gene *28S rRNA* sequences were amplified by Polymerase Chain Reaction (PCR) using Applied Biosystem 2720 thermocycler. The 50ul PCR system was used, including Taq Polymerase Mix 25ul, Mixed primers 2.5ul, DNA template 5ul and Deionized water 17.5ul. All the primers used are universal primers accepted by phylogenetics analysis in freshwater crabs (See Supplementary Table 3, Additional file 1).

After PCR reactions, 1% agarose gel electrophoresis was completed for PCR product quality check. The products with light gel bands were selected, kept at 4 °C and sent to the company for sequencing. The bidirectional sequencing work was done by BGI (Beijing Genomics institution) company, and the platform used was Capillary Sanger Sequencing. After receiving the raw sequencing results, peak figures were checked by SeqMan v7.1.0 to confirm the overall sequencing quality and to assemble the contig sequences.

### Mitogenome sequencing

After searching in NCBI, we found that 11 *Aparapotamon* mitogenome sequences were released. Therefore, we performed the complete mitogenome sequencing on the remaining two new species: *A. binchuanense* and *A. huizeense*. Due to problems in annotation and analysis, we subsequently re-sequenced and obtained another three mitogenome sequences, including one for *A. grahami* and two for *A. gracilipedum.* Five samples were selected, preserved in 95% ethanol and sent to Shanghai Biozeron company for complete next-generation mitogenome sequencing, which was conducted by the Illumina Novaseq platform. High-quality clean data was obtained by removing the adapters and low-quality reads of raw data sets. The de novo assembly of clean data was accomplished by SPAdes v3.10.1 [40] according to the reference mitogenome sequence. All the data were submitted on NCBI with accession numbers: *A. huizeense* (OP355466), *A. binchuanense* (OP355467), *A. grahami* (OM293968), *A. gracilipedum* (ON000286), *A. gracilipedum* (OP526650).

### Phylogenetic analysis

The phylogenetic analysis was conducted based on the concatenated genes (*COX1* + *16S rRNA* + *28S rRNA*) and 13 protein-coding genes (PCGs). The three-gene phylogenetic tree focused on the relationships within the *Aparapotamon* genus. Therefore, multiple sequences of *Aparapotamon* species were chosen for corroboration with morphological results (See Supplementary Table 2, Additional file 1). The sequence alignment was done by MEGA11 muscle algorithm [41] in both pairwise alignment and multiple alignment. SequenceMatrix v1.7.8 [42] was used to connect the multiple gene sequences. The partition homogeneity test was performed using PAUP* [43] to test whether the sequences were suitable for combinational analysis. The conserved regions were chosen by Gblock online [44]. The substitution saturation test was performed by DAMBE7 [45]. The substitution saturation indexes were significantly lower than the threshold value (Iss < Iss.c), indicating that the sequences were little saturated and suitable for phylogenetics analysis.

In this study, for phylogenetic analysis, two methods: Maximum Likelihood (ML) and Bayesian Inference (BI), were chosen to construct the phylogenetic trees. For the three-gene phylogenetic tree, the best substitution model GTR + G + I was selected, and the ML tree was constructed by MAGA11 and tested by bootstrap with 1000 replicates. For the BI tree, MrModeltest2.3 [46] was used to choose the best model GTR + G + I. The BI tree was constructed by MrBayes v3.2.5 [47], which was done by continuously refreshing posterior probabilities. The running generation of four independent Markov Chain Monte Carlo (MCMC) was 20 000 000, sampled every 1000 generations. The first 2000 generations of MCMC chains were thrown off as burn-in. For the phylogenetic tree constructed by three genes, *Longpotamon denticulatum*, *Geothelphusa dehaani*, *Hainanpotamon orientale*, and *Neotiwaripotamon jianfengense* were chosen as the outgroups (See Supplementary Table 2, Additional file 1).

Aiming at exploring evolutionary relationships of the genus *Aparapotamon* in Potamidae and even in Brachyura, the phylogenetic tree based on the 13 PCGs was constructed. Including the mitogenome data of 13 *Aparapotamon* species, 98 mitogenome sequences were selected for constructing the phylogenetic tree, of which 37 were Potamidae (See Supplementary Table 4, Additional file 1). The same procedures were conducted for the 13 PCGs analysis. The best nucleotide substitution model GTR + G + I was selected. The ML tree was constructed by IQ-TREE [48]. The final phylogenetic tree was edited and annotated by the online tool iTOL [49]. For the tree constructed by 13 PCGs, the *Kiwa tyleri* (KY423514) was chosen as the outgroup. The long branch attraction (LBA) was checked and avoided by additionally removing the outgroup and constructing an unrooted tree, which was compared to the original tree [7, 50]. The results showed that LBA has not happened.

### Divergence time estimation

The BEAUti [51] and BEAST v1.8.1 [52] were used for divergence time estimation based on the 13 PCGs. The best evolutionary model, GTR + G + I, was chosen using ModelGenerator [53]. The strict clock was chosen as the molecular clock model with a fixed substitution rate equal to 1. The Yule Process model was set as tree prior. The fossil calibration points were set by the genus *Portunus* 33.9–56.0 Ma, genus *Scylla* 33.9–37.0 Ma, genus *Charybdis* 33.9–35.0 Ma, subsection Raninoida 130.8–133.9 Ma and family Homolidae 145.0–152.1 Ma (Ma: million years ago) [54]. The length of MCMC chain was 20,000,000 generations, with the first 10% discarded as burn-in. The parameters were logged every 2000 generations. The ESS values of each parameter were checked using Tracer1.6 [55], and all the ESS values exceeded 200. The tree was generated by TreeAnnotator v1.8.2 [52], and the divergence time was finally visualized using FigTree v1.4.2.

### Mitogenome annotation and analysis

#### Mitogenome annotation

The complete mitochondrial genomes of all 13 *Aparapotamon* species were annotated and analysed. The MITOS Web Server was used for rough annotation (E-value Exponent = 5, Maximum Overlap = 100, ncRNA overlap = 100) [56]. The PCGs were determined by the open reading frame prediction according to the invertebrate mtDNA translation table. The PCGs and rRNA genes were further confirmed by manually aligning with sequences of closely related species by MEGA11. The genetic spacers and the positions of the start and stop codons were confirmed manually. The MEGA11 was used to analyse nucleotides composition, and the AT-skew, CG-skew were calculated following the formula: AT-skew = (A–T)/(A + T); GC-skew = (G–C)/(G + C).

#### Mitogenome alignment and visualization

The Blast alignment of 13 complete mitogenomes was done by BLAST + v2.11.0 [57], and the alignment results were visualized by BRIG [58]. Further visualization of the particular sequence identity results in detail was done by the mVISTA Web Server [59]. The gene orders of mitogenomes were drawn manually by the online server IBS [60]. All the genes were aligned respectively to observe the occurrence of any genetic changes by Geneious v9.0.2 [61].

### Codon usage analysis

The 13 PCGs of all 13 *Aparapotamon* species were selected and aligned using Geneious v9.0.2. After removing the stop codon, the codon usage analysis and relative synonymous codon usage (RSCU) were calculated using MEGA11. The RSCU results were visualized by the RSCU analysis tool on the online platform JSHYCloud [62]. The nonsynonymous (Ka) and synonymous (Ks) substitution of the 13 PCGs were analysed separately by DnaSP v5.10 [63], and the Ka/Ks ratio was further calculated. The statistical analysis of the Ka/Ks ratio within each gene was carried out and plotted by an online platform [64].

### Transfer RNAs annotation

The websites MITOS Web Server [56] and tRNAscan-SE [65] were used to determine the boundaries of tRNA genes and predict the tRNA secondary structures. The tRNA sequence alignment was done by Geneious v9.0.2. The substitution, deletion and insertion were observed within the genus *Aparapotamon*, and 100% conservation sites were selected. The comparison of tRNA secondary structures between *A. emineoforaminum* and other *Aparapotamon* species was completed manually [66].

### Genetic distance estimation

The genetic distance within each PCG was calculated with the Kimura-2-parameter model by MEGA11. The results were undergone statistics analysis and were visualized as a box plot by the online platform [64]. The genetic distance within each group and between every two groups was calculated based on the 13 PCGs, respectively.

## Results

After performing all the analyses, we integrated the findings, including geographical distributions, morphology, comparative mitochondrial genomics and phylogenetic relationships based on single-gene molecular markers and 13PCGs. Combining all the results, although the bootstrap values between some groups in the single-gene phylogenetic tree are not very high, we believe that the genus *Aparapotamon* can be divided into four groups.

### Geographical distribution

**Fig. 1:**
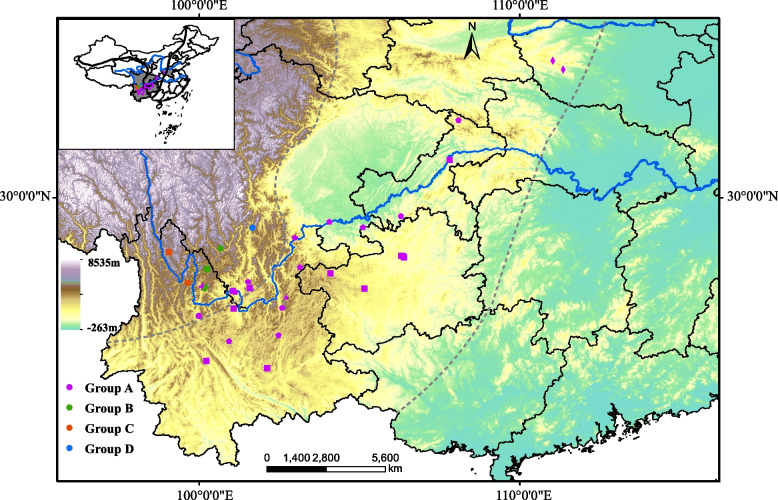
Present distribution of all 13 species in the genus *Aparapotamon*. The map was generated based on the known records and sampling sites in this study. The grey dotted lines indicate the demarcation lines of the three China's terrain ladders. The four different groups are labelled in four different colours. The data used to generate the map was consistent with the *Aparapotamon* species in three-gene phylogenetic trees

According to Fauna Sinica (1999), with Yunnan Province as the dominant distribution area, the genus *Aparapotamon* is widely distributed in 8 provinces in China, including Yunnan, Sichuan, Guizhou, Hunan, Hubei, Chongqing, Shaanxi and Henan [3]. The distribution of all 13 species in genus *Aparapotamon* was mapped (Fig. 1). Based on the relatively independent geographical distributions of the 13 *Aparapotamon* species, the genus was divided into four groups.

Group A, including *A. gracilipedum*, *A. binchuanense*, *A. similium*, *A. huiliense*, *A. huizeense* and *A. grahami*, has a distribution altitude of 500–1900 m above sea level, most of which is southeast of the Yunnan–Kweichow Plateau, and a few species are distributed in the mountains of central plain regions in China. The living environments of group A are mostly under the rocks of narrow mountain streams or mud caves on the shore, with a water depth of 0.02 to 0.3 m, and the water temperature in autumn is 19–21 °C. *A. gracilipedum* lives at an altitude of 1000 m and is found only in Henan Province. *A. binchuanense* is found at an altitude of 1658 m in Dali Bai, Yunnan. *A. similium* is distributed in Yongsheng, Yunnan, living at an altitude of 1800 m. The distribution area of *A. huiliense* is in Huili and Dechang in Sichuan, at an altitude of 1900 m. *A. huizeense* is found in Qujing, Yunnan, at an altitude of 1954 m. Among all the species in group A, *A. grahami* has the broadest distribution, with an altitude of 500–1900 m and crossing multiple mountain ranges and water systems. The distribution areas of *A. grahami* are from the northeastern part of Yunnan and the northwest corner of Guizhou to the southern part of Sichuan, along the Yangtze River system to the northwest of Hunan, the central part of Hubei, and the Zhenping of Shaanxi.

Group B is distributed in the northeast of the Yunnan–Kweichow Plateau, with an altitude of 1500–2700 m and a water depth of 0.02 to 0.5 m, including *A. tholosum*, *A. protinum*, *A. muliense* and *A. arcuatum*. Both *A. tholosum* and *A. protinum* are distributed in Yongsheng, Yunnan. The difference is that *A. tholosum* lives at an altitude of approximately 1500 m, while *A. protinum* lives at an altitude of 1800 to 2400 m. The distribution area of *A. muliense* is in Muli, Sichuan, which is at an altitude of 2200 m. *A. arcuatum* usually does not need a humid environment and is more drought-tolerant than the remaining 3 species of Group B, which usually live under small rocks and caves in mountain streams. *A. arcuatum* is distributed in Ninglang, Yunnan, at an altitude of 2400 to 2700 m. The living environments of *A. arcuatum* are usually below bare rocks and in sand caves next to the shore of mountain streams.

Group C contains 2 species, *A. inflomanum* and *A. molarum*, which are mainly distributed in the middle of the Hengduan Mountain Range at altitudes of 2400 to 2900 m. *A. inflomanum* is distributed at an altitude of approximately 2400 m in Zhongdian, Yunnan, where it lives in mud caves or under rocks in streams. *A. molarum* is a typical species with cold resistance that is distributed in Lijiang, Yunnan, at an altitude of 2400 to 2900 m. It usually lives under the rocks of streams near snow-capped mountains at a water depth of 0.3 to 1 m and a temperature of 9–10 °C in summer.

Group D includes *A. emineoforaminum*, which lives in Mianning and Xichang, Sichuan Province, at an altitude of 1600 to 1800 m, where it inhabits streams and aquatic grasses at a water depth of 0.1 m.

### Morphological classification

In this study, the overall morphological characteristics of genus *Aparapotamon* were: All individuals were medium in size. Carapace trapezoidal, dorsal surface slightly convex, regions defined. External orbital angle round, separated from anterolateral margin, postorbital cristae convex, postfrontal lobe prominent. Cervical groove shallow, H-shaped groove distinct. Epibranchial tooth distinct, especially in female specimens. Third maxilliped exopod without flagellum. Ambulatory legs slender. Male pleon broad triangular, telson triangular, apex rounded. Vulva ovate. G1 claviform, slender, beyond pleonal locking tubercle, G2 basal segment ovate, tip of terminal segment round.

**Fig. 2:**
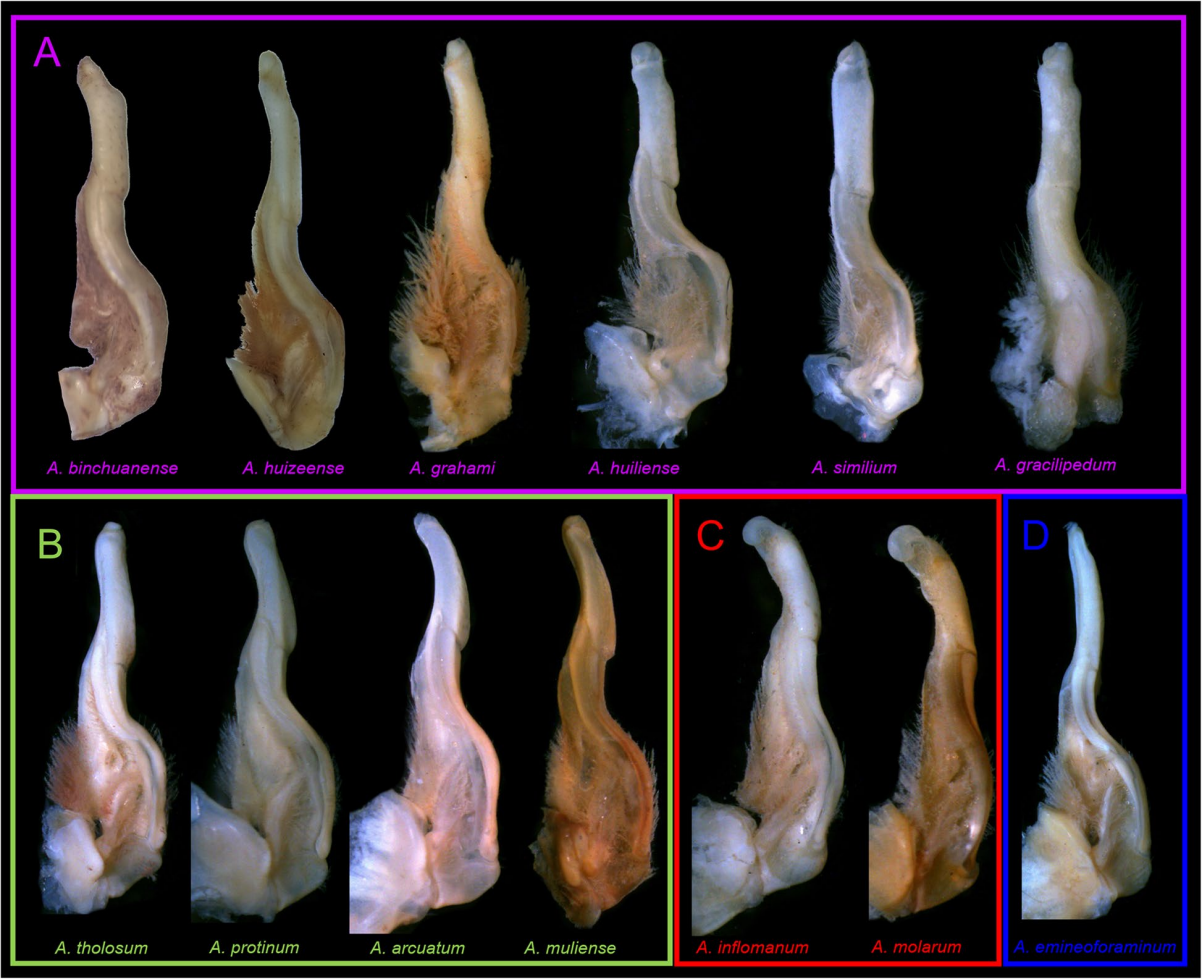
G1 morphology of 13 species holotypes in genus *Aparapotamon*. The names of species in the same group are in the same colour

During the morphological observation of specimens of genus *Aparapotamon*, it was found that the G1 morphology of holotypes were diverse among different species and can be divided into four groups (Fig. 2). For group A: the terminal segments of G1s relatively straight, and dorsal lobe variably developed inwards. For group B: dorsal lobe of terminal segments of G1s variably developed upwards; in *A. tholosum and A. muliense,* terminal segments of G1s arc-shaped. For group C: distal end of G1s disc-shaped. For group D: the terminal segment of G1 very slender, tapering distally.

### Phylogenetic inference

**Fig. 3:**
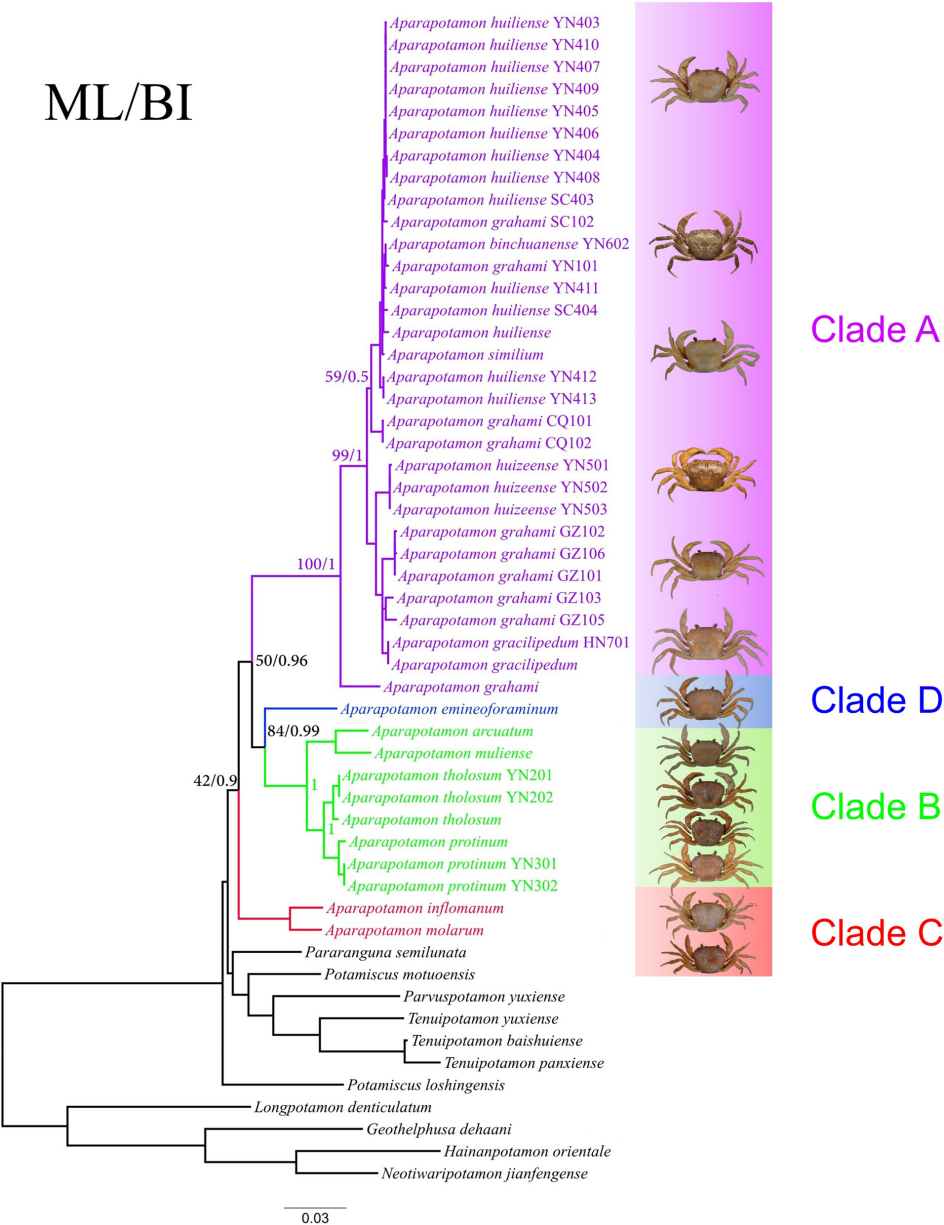
Maximum Likelihood (ML) tree/Bayesian Inference (BI) tree constructed by concatenated genes (*COX1* + *16S* *rRNA* + *28S rRNA*). The trees built by ML (See Supplementary Fig. 1, Additional file 2) and BI (See Supplementary Fig. 2, Additional file 2) have been combined. The numbers at the internodes are the maximum likelihood bootstrap proportions and bayesian inference posterior proportions. The positions of the species morphology in the figure correspond to the species name on the phylogenetic tree. Information on the sequences and numbers is provided (See Supplementary Table 2, Additional file 1)

**Fig. 4:**
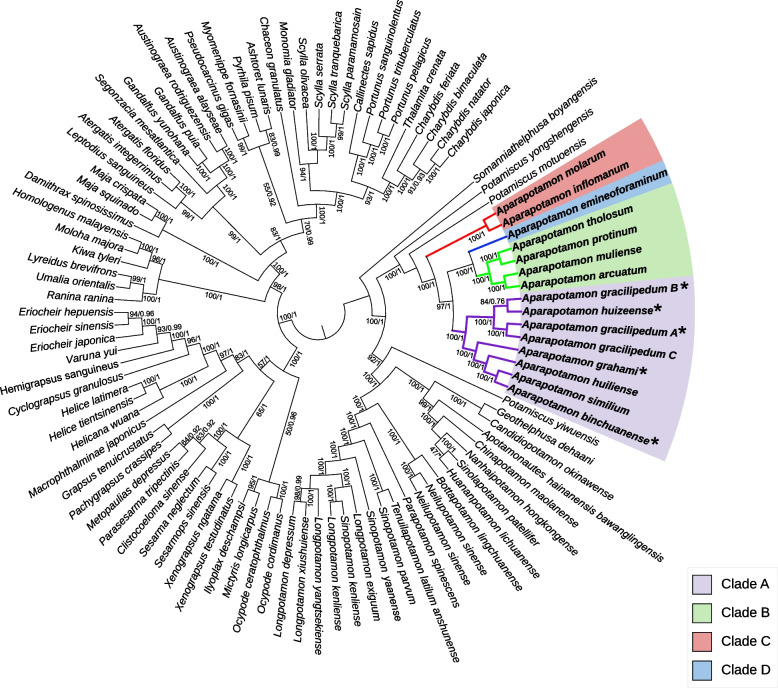
Maximum Likelihood tree/Bayesian Inference tree constructed by 13 PCGs. The trees built by ML (See Supplementary Fig. 3, Additional file 2) and BI (See Supplementary Fig. 4, Additional file 2) have been combined. The numbers at the internodes are maximum likelihood bootstrap proportions and bayesian inference posterior proportions. The branch lengths have been ignored to make the tree more orderly. The newly sequenced species are marked with the star after the species name. For *A. gracilipedum*: A (ON000286); B (OP526650); C (MZ350905)

The topological structures of maximum likelihood (ML) and Bayesian inference (BI) phylogenetic trees revealed the relationships between *Aparapotamon* species (Fig. 3) and the evolutionary position of the genus *Aparapotamon* in the family Potamidae (Fig. 4). According to the results, including *A. molarum* and *A. inflomanum*, clade C branched first. *A. tholosum*, *A. protinum*, *A. muliense* and *A. arcuatum* grouped together to form clade B. Then, clade B and clade D (*A. emineoforaminum*) formed one branch, which was further grouped together with six species in clade A. Regarding the phylogenetic relationships of *Aparapotamon* in Potamidae, the genus clustered with *Potamiscus motuoensis* and *Potamiscus yongshengensis*. The topological structures of the ML and BI trees were nearly the same, and most of the branches had a high confidence level, which indicated the credibility of the results. The species classification results from phylogenetic analysis were the same as those obtained from morphological examination and geographical distribution patterns. The verification from all of the above analysis results increased the credibility of our conclusion of four groups, through which the taxonomy of the genus *Aparapotamon* was confirmed.

The genetic distance based on 13 PCGs was calculated under the Kimura 2-parameter model (See Supplementary Table 5, Additional file 1). Overall, the mean genetic distance within groups was significantly shorter than the genetic distance between groups. Group A had the shortest intragroup mean distance (0.04), followed by group C (0.05), and the within-group mean distance of group B was the longest (0.07). According to the results of the mean genetic distance between groups, group B and group D had the shortest genetic distance, at only 0.103, which verified the relatively close relationship between groups B and D compared to other groups. The longest genetic distance was observed between group A and group B, which was 0.352. There was no within-group mean distance in group D, as only one species, *A. emineoforaminum,* was classified into group D.

### Divergence time estimation

According to the divergence time estimation results (See Supplementary Fig. 5, Supplementary Fig. 6, Additional file 2), genus *Aparapotamon, Potamiscus motuoensis*, and *Potamiscus yongshengensis* were clustered on a large branch, the estimated divergence time of which was 42.69 Ma (95% credibility interval = 40.75–44.54 Ma).

The estimated divergence time of genus *Aparapotamon* was 18.1 Ma (95% credibility interval = 17.11–19.09 Ma). The divergence time of group C was approximately 16.67 Ma (95% credibility interval = 15.77–17.59 Ma), which was branched first in the genus *Aparapotamon*. Groups D and B were branched with the estimated divergence time of 11.82 Ma (95% credibility interval = 11.01–12.64 Ma). Species in group B were further branched in approximately 7.13 Ma (95% credibility interval = 6.61–7.69 Ma). The divergence time between groups A and B, D was approximately 15.56 Ma (95% credibility interval = 14.65–16.45 Ma). The *A. gracilipedum* and *A. huizeense* clustered together with an estimated divergence time of 2.5 Ma (95% credibility interval = 2.26–2.76 Ma). The other four species in group A were clustered together in about 3.08 Ma (95% credibility interval = 2.82–3.37 Ma).

According to the apparent divergence time of the genus *Aparapotamon*, group C branched first, followed by group B and group D, which clustered relatively closely. The estimated divergence time of species within group A was the latest among the four groups. Considering all these findings, including morphology, geographical distribution, and phylogenetic relationships, our conclusion of four groups was confirmed. We then aimed to find more evidence based on comparative mitochondrial genomics and to study the adaptive evolution of the *Aparapotamon* genus.

### Mitogenome composition

The complete circular mitogenomes of the genus *Aparapotamon* are between 15,780 bp and 19,482 bp in size, the shortest of which is observed in *A. grahami* (MZ350906), and the longest is in *A. molarum* (Table 1). All 13 *Aparapotamon* species exhibit the same mitogenome composition structures. The coding region of the mitogenomes contains 37 typical genes in total, including 13 protein-coding genes, 22 tRNA genes and 2 rRNA genes. The mitogenomes exhibited high AT compositions, with an average of 73.4%. Among the four groups, *A. molarum* had the highest AT content (74.6%), and group A showed a higher AT content (73.6% on average) than the other three groups. Additionally, species in the same groups showed similar base compositions, which also proves our classification. The average AT skew of the complete mitogenomes was -0.03, and the average GC skew was -0.34, showing slight TA bias and CG bias. The degree of CG bias was significantly higher than that of TA bias. The values of AT skew and GC skew were consistent with those of published Potamidae mitogenome compositions [35, 54].

**Table 1:** Characteristics of the mitochondrial genomes of the genus *Aparapotamon*

Group	Name	GenBank	Length (bp)	AT%	AT skew	GC skew	PCG length (bp)	No. sense codons	PCG AT%
A	*A. similium*	MZ350912	18043	73.9	-0.03	-0.34	11148	3705	71.34
	*A. huiliense*	MZ350907	18186	73.9	-0.03	-0.34	11148	3705	71.15
	*A. grahami*	MZ350906	15780	72.8	-0.03	-0.31	11148	3705	71.21
	A. grahami*	OM293968	17951	73.7	-0.03	-0.34	11148	3705	71.01
	A. gracilipedum*	ON000286	17988	73.7	-0.03	-0.34	11148	3705	71.00
	A. gracilipedum*	OP526650	17969	73.7	-0.03	-0.34	11149	3706	71.07
	*A. gracilipedum*	MZ350905	16894	73.2	-0.03	-0.32	11145	3704	70.79
	A. binchuanense*	OP355467	17995	74.0	-0.03	-0.33	11148	3705	71.42
	A. huizeense*	OP355466	17997	73.8	-0.03	-0.34	11148	3705	71.08
B	*A. muliense*	MZ350910	19212	72.9	-0.03	-0.35	11148	3705	69.62
	*A. arcuatum*	MZ350903	19128	72.8	-0.03	-0.34	11148	3705	69.52
	*A. tholosum*	MZ350914	16605	72.2	-0.03	-0.36	11151	3706	70.00
	*A. protinum*	MZ350911	17907	72.3	-0.03	-0.36	11151	3706	69.67
C	*A. inflomanum*	MZ350908	16287	73.0	-0.04	-0.31	11151	3706	70.64
	*A. molarum*	MZ350909	19482	74.6	-0.03	-0.34	11151	3706	70.51
D	*A. emineoforaminum*	MZ350904	19432	73.2	-0.02	-0.34	11151	3706	69.72

The newly sequenced species in this study are marked with the star (*)

**Fig. 5:**
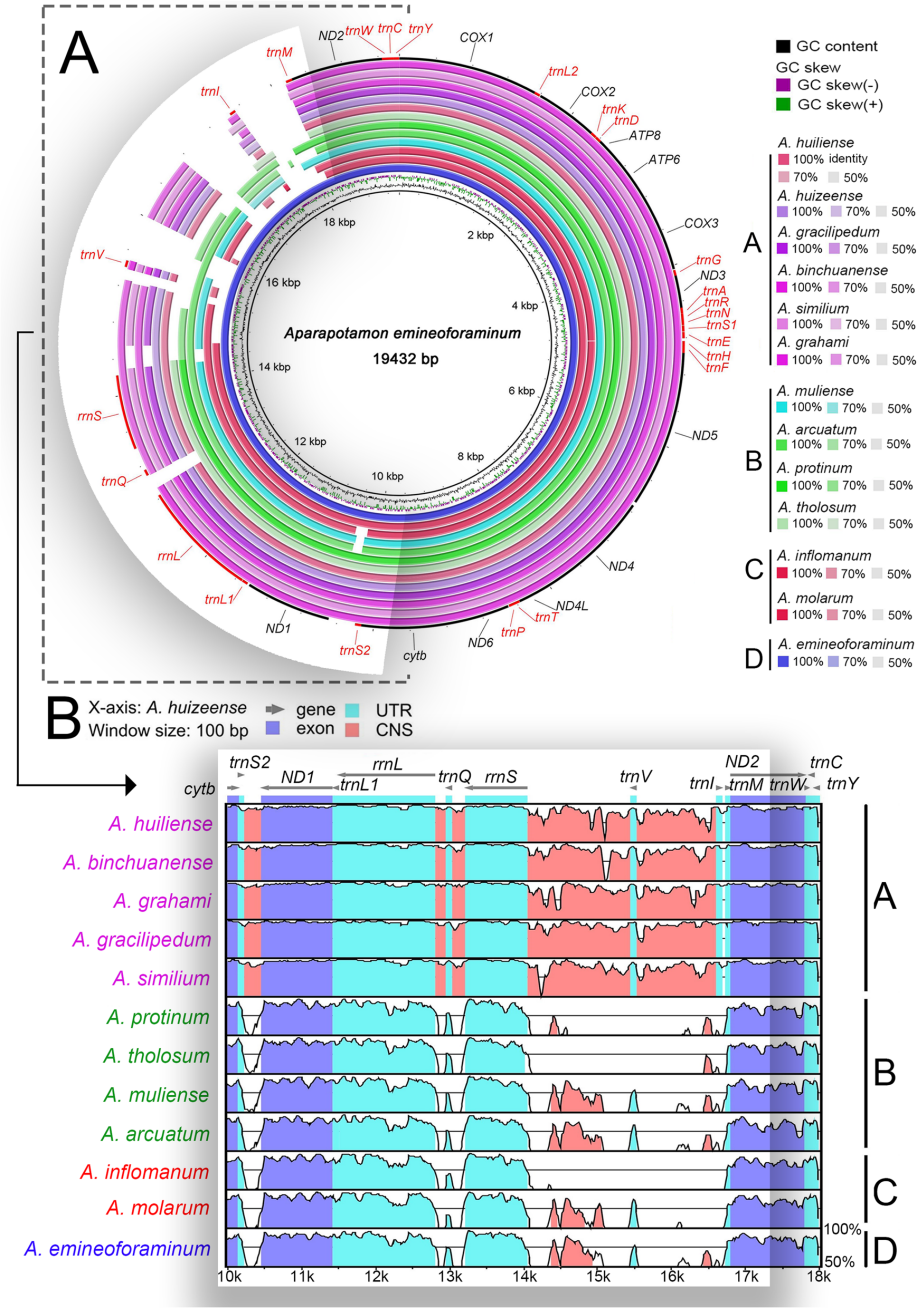
(**A**) Blast comparison of 13 representative *Aparapotamon* mitogenomes. The comparison has been made with the *A. emineoforaminum* (group D) as the reference species. In the outermost ring, the protein-coding genes are labelled in black, and other genes are labelled in red. The species in the same group are in similar colours. The representative sequences used are *A. grahami* (OM293968) and *A. gracilipedum* (OP526650). (**B**) Major intragroup sequence identity variation visualized by blast alignment. The alignment was done against *A. huizeense* in group A, from 10,000 bp to 17,997 bp. The names of species in the same group are in the same colour

After performing multiple alignment to the complete mitogenomes of 13 *Aparapotamon* species, apparent sequence homology within the genus *Aparapotamon* was found (Fig. 5A). Specifically, relatively high and stable sequence identity was found in the protein-coding regions, which were nearly all more than 75% between each pair of *Aparapotamon* species. The mitogenome sequence identity within each group was significantly higher than the sequence identity across different groups. This indicated that species within each group are more homologous, which also verifies our four-group classification results based on morphological identification, geographical distribution and phylogenetic analysis. Taking *A. huizeense* as an example, by blast alignment, a significantly higher identity between *A. huizeense* and the other 5 species in group A was observed (Fig. 5B). The relatively high intragroup sequence identity was reflected not only in the gene coding regions but also in the noncoding regions (CNS). Such regularities were also found in group B and group C.

### Mitogenome arrangement

During alignment, it was found that in the genus *Aparapotamon*, the genes encoded by the heavy strand were more compact, while the genes encoded by the light strand, especially the genes near the putative control region and genes encoded at the junction of the heavy and light strands, were loosely arranged and had a relatively large intergenic spacer. The length of gene overlap was relatively conserved. Seven gene overlaps, 20 bp in total, occurred in all *Aparapotamon* species, which included the two typical 7 bp overlaps between *ATP8* and *ATP6* and between *ND4* and *ND4L*. For genes *ATP8* and *ATP6*, the sequence of the overlapping region was ATTATAG, which included the termination codon TAG of *ATP8* and the initiation codon ATT of *ATP6*. For *ND4* and *ND4L*, the overlapping sequence was TTAACAC, which was GTGTTAA in the complementary strand, including the termination codon TAA of *ND4L* and the initiation codon GTG of *ND4*. These two overlaps are typically found in other invertebrate species [67]. In these two overlapping regions, two groups of genes are transcribed in different reading frames, which can increase transcriptional efficiency. In the species *A. gracilipedum* (MZ350905), the duplication of these two short nucleotide sequences was observed by alignment. Other overlaps observed in all *Aparapotamon* species included a 2 bp overlap between *ND2* and *tRNA-Trp* and 1 bp overlaps between *ATP6* and *COX3*, *ND3* and *tRNA-Ala*, *tRNA-Arg* and *tRNA-Asn*, and *ND6* and *cytb*. In addition, a 1 bp overlap between *tRNA-Trp* and *tRNA-Cys* was observed in 11 *Aparapotamon* species, excluding *A. inflomanum* and *A. molarum*, which could be a feature specific to these two species in group C. A 1 bp overlap between *tRNA-Thr* and *tRNA-Pro* was additionally observed in *A. emineoforaminum*.

**Fig. 6:**
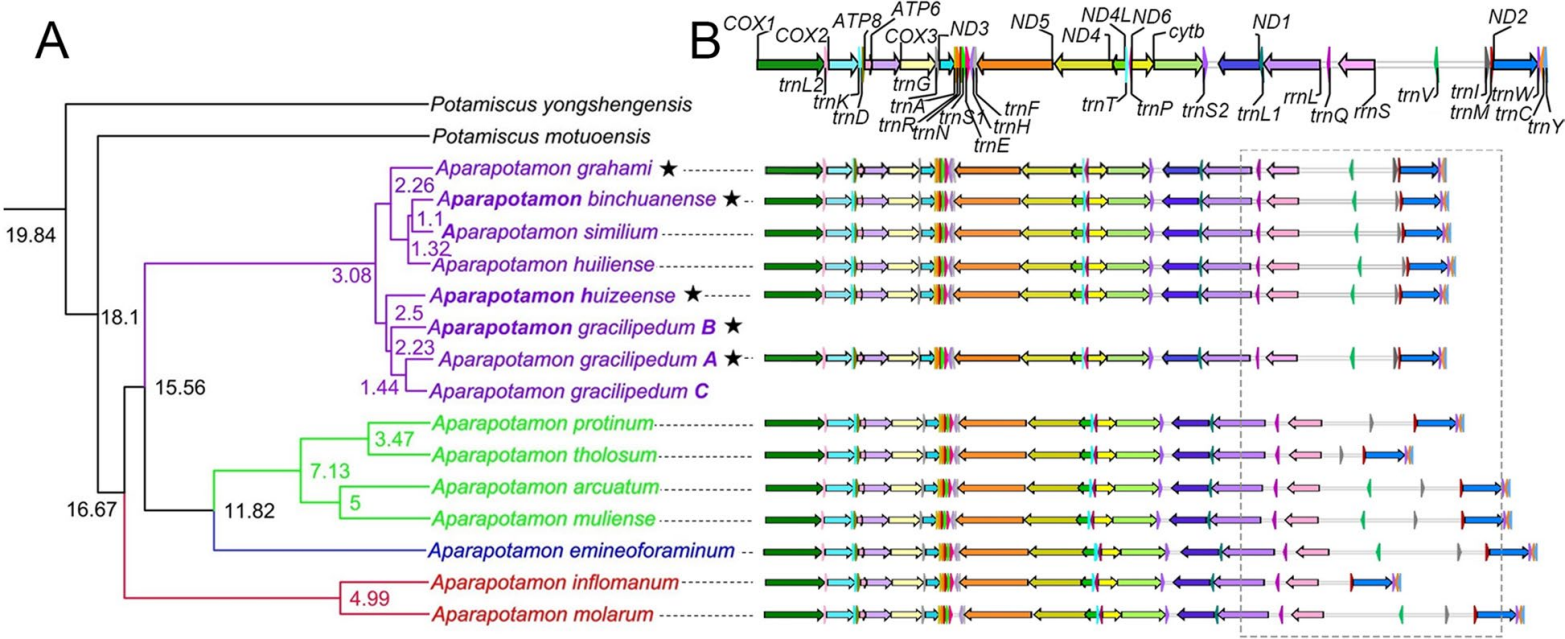
**A** Divergence time estimation of genus *Aparapotamon*.The digital numbers behind the branch indicated the estimated species divergence time (Ma: million years ago). The species sequenced in this study are marked by a star after the name. For *A. gracilipedum*: A (ON000286); B (OP526650); C (MZ350905). **B** The linear genome arrangements of 13 species in the genus *Aparpatamon*. The grey dotted frame indicates the region with great variety, including the specific arrangement of *tRNA-Ile* in group A

Compared to the gene arrangement of the ancestor of Brachyura, most of the gene arrangements between the *Aparapotamon* genus and Brachyura were similar [54, 68]. Two gene rearrangements occurred in the genus *Aparapotamon* compared to the ancestor of Brachyura, which all occurred in the light strand. *tRNA-Val* was rearranged after *12S rRNA* in the middle of the longest noncoding region in the mitogenomes. *tRNA-Gln* was rearranged between the *16S rRNA* and *12S rRNA* genes*.* During the annotation, the *tRNA-Val* of *A. tholosum*, *A. protinum* and *A. inflomanum* and *tRNA-Ile* of *A. inflomanum* could not be identified by online server or annotated by alignment with closely related species. It was speculated that some problems occurred during sequencing and assembly due to the special locations of these two *tRNA* genes, which were near the unstable putative control region. Combined with the four-group classification results obtained by the phylogenetic and divergence time estimation analysis (Fig. 6A), the genome arrangements within the genus *Aparapotamon* have been compared (Fig. 6B). Except for sequences that were not fully annotated, no gene rearrangement was observed within the genus *Aparapotamon*. The genes of all 13 *Aparapotamon* species were in the same order. The variety of gene arrangement was mainly concentrated in the large noncoding region, in which the locations of *tRNA-Ile* in the genus *Aparapotamon* were different between groups. The six species of group A showed the same arrangement patterns, and the intergenic region between the *tRNA-Ile* and *tRNA-Met* genes ranged from 43 to 67 bp. However, in other *Aparapotamon* species, the noncoding region between the *tRNA-Ile* and *tRNA-Met* genes was significantly larger, from 526 bp (*A. tholosum*) to 1158 bp (*A. muliense*) in length.

### Overview of PCGs in the genus *Aparapotamon*

**Fig. 7:**
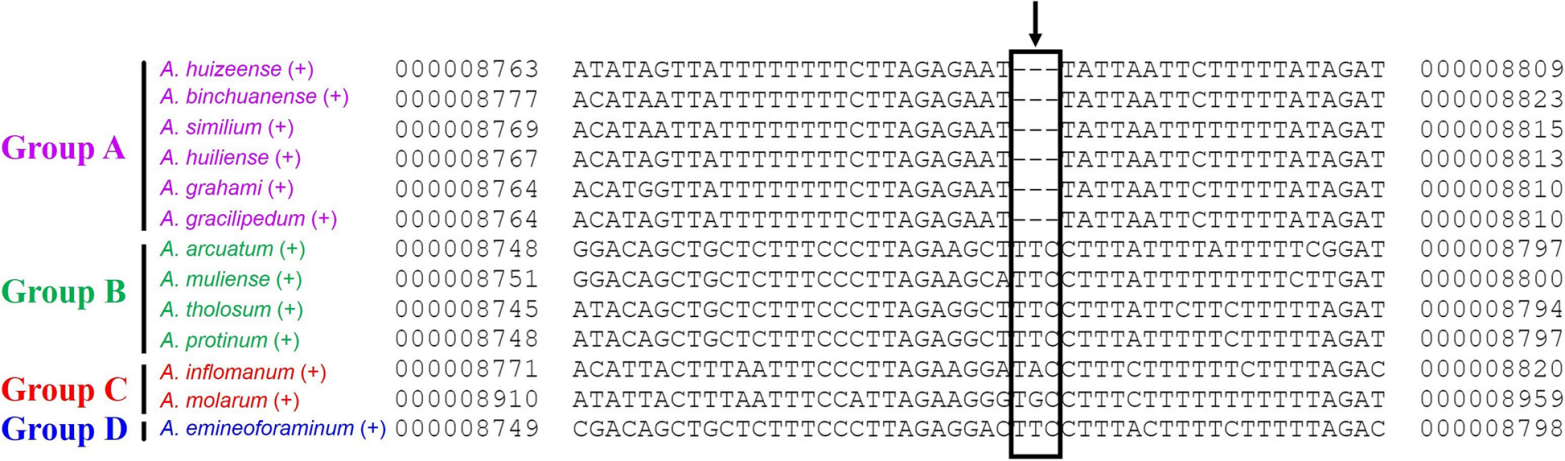
The codon loss in gene *ND6* in all six species in group A. The codon loss can be observed in the same position 416 of gene *ND6* in group A species after blast alignment

The length of 13 PCGs in the genus *Aparapotamon* ranged from 11,145 bp to 11,151 bp. By pairwise and multiple alignment, we found that there were some codon loss conditions, which were the reason for the variation in PCG length. All six species in group A showed the loss of genetic code at position 416 of the *ND6* gene (Fig. 7). In this location, the same codon, TTC, was used by group B and group D, while in group C, purines (A/G) were chosen as the secondary base instead of thymidine. In the *ND2* gene, codon loss was also observed in the species *A. gracilipedum* (MZ350905), *A. muliense*, and *A. arcuatum* during sequence alignment. The codon loss was at the same location in the species *A. muliense* and *A. arcuatum*, which demonstrated the common characteristics of the two species in group B. The rationality of the classification was also confirmed from the perspective of PCGs. In *A. gracilipedum* (OP526650), due to the change in the termination codon from TAA to a single T in gene *COX2*, one more codon was observed at the end of *COX2* compared to the others from species in group A. In the relatively conserved protein-coding region, *A. gracilipedum* exhibited noteworthy instability compared to the other *Aparapotamon* species.

**Fig. 8:**
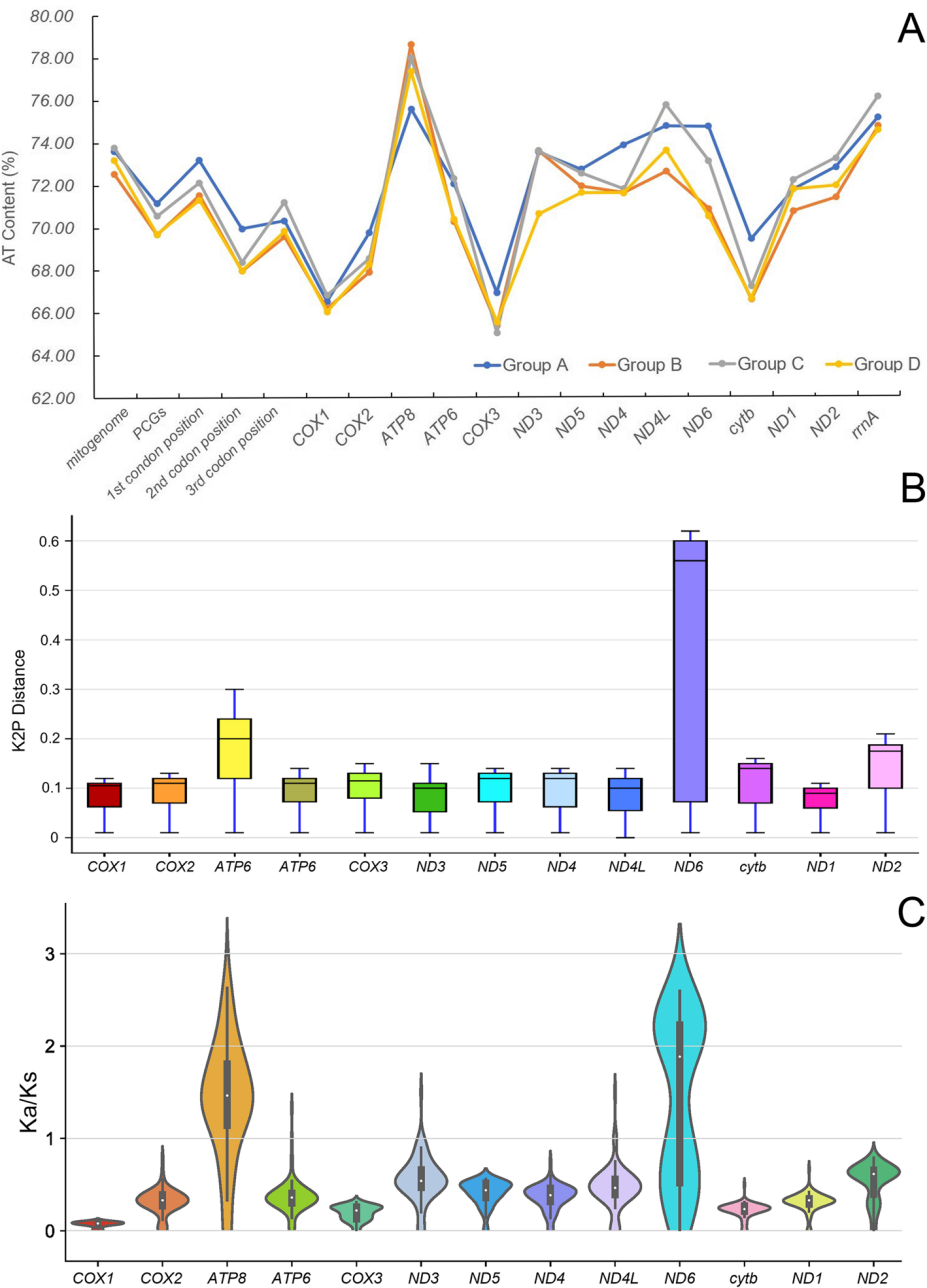
The informatics analyses of species in genus *Aparapotamon*. **A** The average mitogenome AT content of the four groups in the genus *Aparapotamon*. **B** The box plot for the pairwise interspecific Kimura-2-parameter distance of 13 PCGs in all 13 species in genus *Aparapotamon*. **C** The pairwise nonsynonymous and synonymous ratio (Ka/Ks) of 13 PCGs in all 13 species in the genus *Aparapotamon*

The composition of 13 PCGs also exhibited a high AT content. Compared to that of the complete mitogenome, the AT contents of the 13 PCGs were lower, with an average of 70.61%. Among all groups, the AT contents of the six *Aparapotamon* species in group A were the highest (71.12% on average), followed by group C (70.58% on average), and the AT contents in groups B and D were low (Fig. 8A). This suggested that species in group A have a higher preference for bases A and T, which to some extent may also reflect the species' usage preference for certain codons. The lower AT content in groups B and D (69.70% and 69.72%, respectively, on average) reflected the relatively close relationships between *A. emineoforaminum* in group D and group B. Among all 13 PCGs detected, the *ATP8* gene contained the highest AT content (77.38% on average), and *COX3* had the lowest AT content (65.71% on average). Among the genes in the four groups, the AT content of five PCGs displayed significant variation, including genes *ND6*, *ND4L*, *ATP8*, *ND3*, and *cytb,* with the highest variation in *ND6* (70.48%-74.71%). Except for the *ND3*, *ND4L* and *ND1* genes, the PCGs in group B and group D showed almost identical AT contents, which also verified the close relationship between the two groups.

The pairwise Kimura-2-parameter (K2P) distance within each of the 13 PCGs was calculated (Fig. 8B). In the results, the greatest interspecific K2P distance and variation degree was observed in the *ND6* gene*,* with an overall mean K2P distance of 0.36 and a variation degree ranging from 0.01–0.62, followed by *ATP8* and *ND2*, with an overall mean interspecific K2P distance of 0.18 and 0.14, respectively. This demonstrated that these three genes have maintained higher rates of genetic variation during evolution than the other PCGs in the genus *Aparapotamon*. *ND1* exhibited the lowest mean K2P distance, followed by *ND4L*, *ND3* and *COX1*, which indicated that these genes were highly conserved. The mean interspecific K2P distances of these genes were 0.076, 0.085, 0.086 and 0.0861, respectively.

The nonsynonymous to synonymous substitution ratios (Ka/Ks) of all 13 species in the genus *Aparapotamon* were calculated (Fig. 8C), through which the selection pressure could be measured. According to the results, the Ka/Ks values of eight genes were all < 1, including *COX1*, *COX2*, *COX3*, *ND5*, *ND4*, *cytb*, *ND1* and *ND2*, which underwent purifying selection and were relatively stable and conserved during evolution, with vitally important functions [36]. Among them, *COX1* showed the lowest Ka/Ks ratio, with an average of 0.07, which means that there were few amino acid substitutions in *COX1*. In three genes, *ATP6*, *ND3* and *ND4L*, the Ka/Ks values were < 1 with a few exceptions, indicating that in most species, these genes have experienced purifying selection. For *ATP6*, the Ka/Ks between the two species in group A, *A. similium* and *A. binchuanense,* was 1.33, which means that this gene has been subjected to positive selection during the evolutionary process of these two species, potentially providing evidence of speciation, as in the later phylogenetic analysis, the close relationship between these two species was verified. The same condition also occurred for the *ND3* and *ND4L* genes between *A. huiliense* and *A. grahami* in group A. The Ka/Ks value in *ND3* between these two species was 1.5, and that in *ND4L* was also 1.5, which means that these two genes have been exposed to positive selection. Genes *ND6* and *ATP8* have an average Ka/Ks ratio > 1, which demonstrates that these two genes have experienced positive selection.

In the pairwise K2P distance and Ka/Ks calculation, the *ATP8* and *ND6* genes showed great genetic variation and were relatively nonconserved compared to other protein-coding genes in the mitogenomes within the genus *Aparapotamon*. Additionally, the AT content of *ATP8* was the highest among all the PCGs. In addition, in the six species in group A, codon loss was found in the *ND6* gene, which resulted in the shorter PCG length of group A. Therefore, it can be speculated that the *ATP8* and *ND6* genes have high mutation rates and play important functions in the adaptive evolution of the genus *Aparapotamon*.

### Codon usage

In the codon usage for 13 PCGs of genus *Aparapotamon*, ATG was mainly used as the initiation codon and TAA as the termination codon. For the initiation codons, eight genes (*COX1*, *COX2*, *ATP8*, *COX3*, *ND5*, *ND4L*, *cytb*, *ND2*) used ATG as the initiation codon. Two genes, *ATP6* and *ND3,* used ATT as the initiation codon. GTG was used by two genes, *ND4* and *ND1*, except for *A. molarum* (group C), which used ATG as the initiation codon for gene *ND4*. Only gene *ND6* used the initiation codon ATA. For the termination codons, eight genes (*COX2*, *ATP6*, *COX3*, *ND4*, *ND4L*, *ND6*, *ND1*, *ND2*) used TAA as the termination codon, except *A. molarum* used TAG for gene *COX2*. Two genes *ATP8* and *ND3* used TAG as the termination codon. Genes *COX1*, *ND5*, and *cytb* used single incomplete T as the termination codon, which can form the complete TAA terminator at the post-transcriptional level by polyadenylation, adding poly A tail at the 3’ end of mRNA.

**Fig. 9:**
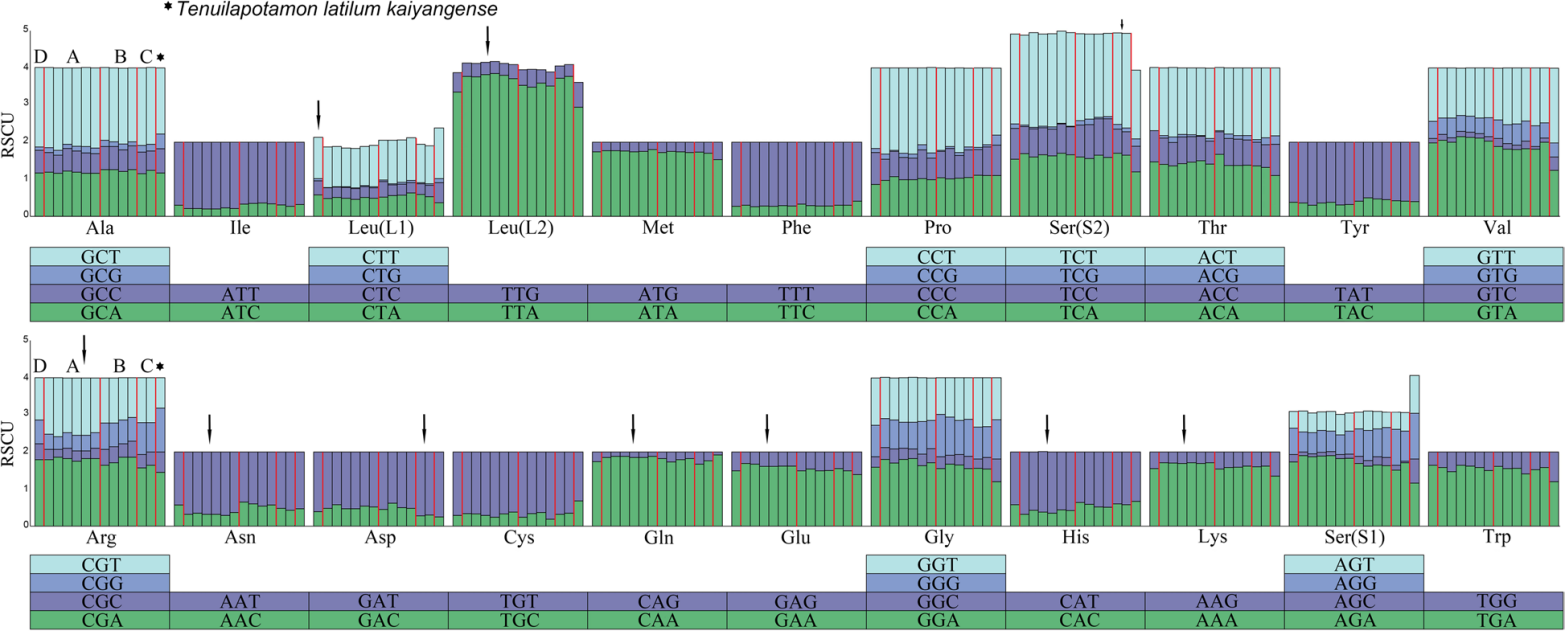
The RSCU results of genus *Aparapotamon* and *Tenuilapotamon latilum kaiyangense* (MW788029). From the left to right: group D: *A. emineoforaminum*; group A: *A. similium*, *A. huiliense*, *A. grahami* (OM293968), *A. gracilipedum* (ON000286), *A. binchuanense*, *A. huizeense*; group B: *A. muliense*, *A. arcuatum*, *A. tholosum*, *A. protinum*; group C: *A. molarum*, *A. inflomanum*; *Tenuilapotamon latilum kaiyangense* was chosen as an outgroup for comparison, which is marked by a star. The codon usage, which can show between-group differences, is labelled with arrows

The number of sense codons in PCGs of the genus *Aparapotamon* ranged from 3704 to 3706. The codon preference results also reflected an AT preference, and at the same time, the end base of synonymous codons was clearly biased towards base A or T. Within the genus *Aparapotamon*, a slight difference in relative synonymous codon usage (RSCU) values between the four groups was observed (Fig. 9). When encoding the amino acid *Leu (L2)*, group A and group C showed an obvious preference for the codon TTA compared to group B and group D. In the selection of the third base of codons for *Arg*, *Asn* and *His*, base T was more frequently used in the six species in group A than in the other three groups. When encoding *Ser* (*S2*), *Gln*, *Glu* and *Lys*, group A had a significantly higher preference for bases A and T than the other three groups in the selection of the third base. In terms of codon usage frequency, group D (*A. emineoforaminum*) exhibited similarities with the four species in group B, which also helped verify the close relationship between groups B and D. Due to similarity in geographical distributions, *Tenuilapotamon latilum kaiyangense* (MW788029) was chosen as an outgroup for comparison.

### Transfer RNAs

A total of 22 tRNA genes were detected in each *Aparapotamon* species, the length of which ranged from 61 to 73 bp. All tRNA genes were predicted to fold into the typical cloverleaf secondary structures except the dihydroxyuridine arm (DHU arm) of *tRNA-Ser (S1)*, which cannot form the typical stem-loop structure. The lack of the DHU arm of this tRNA is commonly found in metazoan mitogenomes and even in those of mammals and insects [69, 70].

**Fig. 10:**
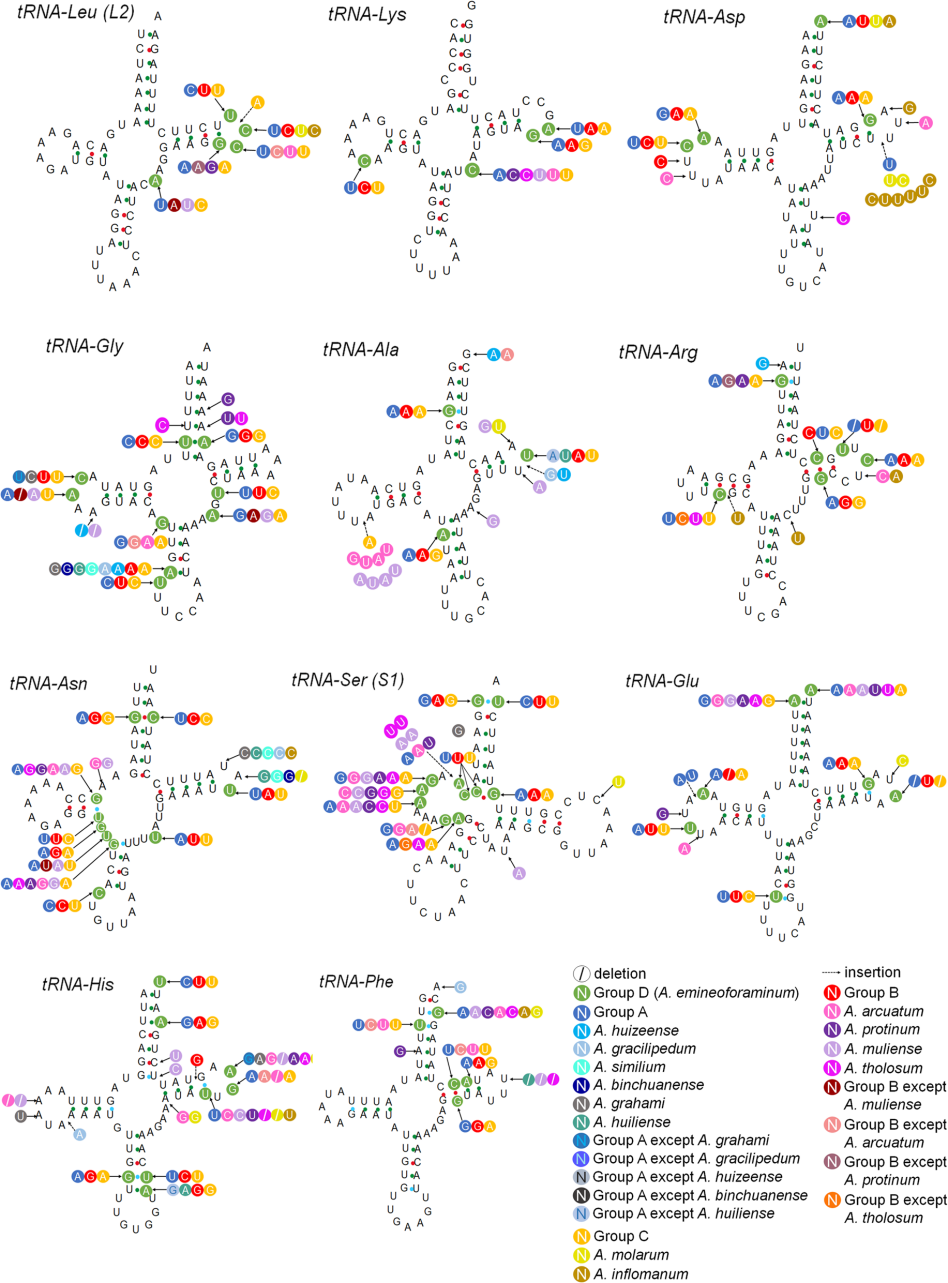
The predicted secondary structure of tRNA genes, from *tRNA-Leu (L2) *to *tRNA-Phe*. The nucleotide substitution pattern of tRNA genes in genus *Aparapotamon* has been exhibited with the reference species *A. emineoforaminum* in group D. Any insertion or deletion has been noted. Similar colours are used to label species in the same group

**Fig. 11:**
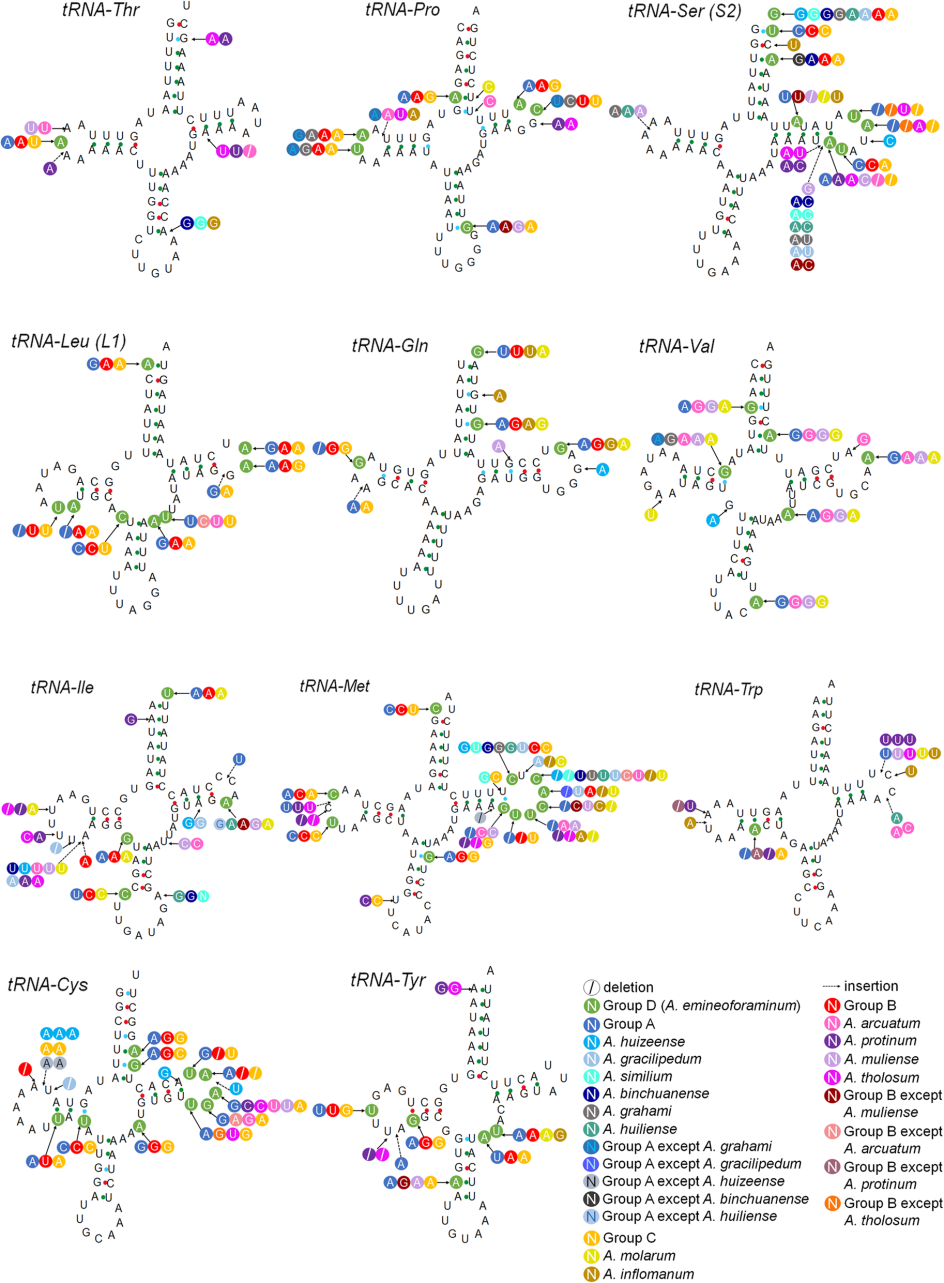
The predicted secondary structure of tRNA genes, from *tRNA-Thr* to *tRNA-Tyr*. The nucleotide substitution pattern of tRNA genes in genus *Aparapotamon* has been exhibited with the reference species *A. emineoforaminum* in group D. Any insertion or deletion has been noted. Similar colours are used to label species in the same group

The predicted secondary tRNA structures of all *Aparapotamon* species were compared with that of *A. emineoforaminum* as a reference, which was the only species in group D (Figs. 10, Fig. 11). Overall, in all 13 *Aparapotamon* species, the acceptor arm was 7 bp in length, the end of which could bind amino acids. The TφC loop ranged from 2 bp-7 bp. The anticodon loop ranged from 7 bp-9 bp and functions to recognize and bind to the codons of mRNA. The length of the D-loop ranged from 3 to 10 bp. After the alignment of tRNA gene sequences, 100% conserved sites among the 13 *Aparapotamon* species was observed, and the proportion was calculated (Table 2). *tRNA-Lys* was the most conserved during evolution among the 22 tRNA genes in the genus *Aparapotamon*, the 100% conserved site proportion of which was 0.94. Then, the second most conserved tRNA genes were *tRNA-Leu (L2)* and *tRNA-Trp*, whose proportion was 0.92. Other tRNA genes that had a proportion > 0.9 were t*RNA-Thr* and *tRNA-Gln*. Most of the tRNA genes had a proportion between 0.8 and 0.9. The 100% conserved site proportions of *tRNA-Arg*, *tRNA-Gly* and *tRNA-His* were < 0.8, and the tRNA gene that had the lowest 100% conserved site proportion was *tRNA-Met* (0.75), which was the least conserved tRNA gene.

**Table 2:** Overview of the comparison results of tRNA genes in the genus *Aparapotamon*

tRNA	Alignment length (bp)	No. 100% conserved sites	Proportion of 100% conserved sites	Sites with intergroup differences	Mutation sites of individual species (Indel/substitution)
*Cys*	65	52	0.8	11	7
*Asn*	66	53	0.8	10	6
*Ser (S1)*	66	53	0.8	10	9
*Leu (L1)*	64	56	0.88	9	1
*Gly*	63	50	0.76	8	9
*Ser (S2)*	69	57	0.83	8	10
*Glu*	68	59	0.87	7	5
*Pro*	62	53	0.85	7	8
*Leu (L2)*	64	59	0.92	6	4
*Arg*	61	47	0.77	6	6
*His*	65	51	0.78	6	11
*Val*	72	63	0.88	6	4
*Met*	71	53	0.75	6	12
*Tyr*	63	56	0.89	6	4
*Gln*	68	61	0.9	5	6
*Ile*	66	55	0.83	5	10
*Lys*	65	61	0.94	4	1
*Asp*	65	56	0.86	4	7
*Phe*	68	59	0.87	4	6
*Ala*	63	55	0.87	3	6
*Thr*	64	58	0.91	1	5
*Trp*	63	58	0.92	1	6

The tRNAs are arranged in descending order of the number of sites, which reflects intergroup differences

During the comparison, any mutations such as insertions, deletions and substitutions were noted. Importantly, some changes reflecting intragroup differences were observed. In total, 133 sites in the tRNA genes showed differences at the group level, which supported the four-group classification of the genus *Aparapotamon* proposed in this study. Some mutation sites that occurred in individual species were also counted, which included 143 sites in total. Most mutations occurred in the D-loop and TφC loop, among which base substitution was the most common type of mutation. In addition, G-U mismatch occurred most commonly and usually in the stem region of the tRNA secondary structures. Due to the problems in sequencing and assembly, the *tRNA-Val* of *A. tholosum*, *A. protinum* and *A. inflomanum* and *tRNA-Ile* of *A. inflomanum* could not be annotated.

### Ribosomal RNAs

The length of *16S rRNA* and *12S rRNA* in genus *Aparapotamon* ranged from 2119 bp (*A. huiliense*) to 2150 bp (*A. protinum*). The AT content on average of the two rRNA genes was 75.12%, which was higher than the average AT% of the complete mitogenome and 13 PCGs. It should be noticed that group C exhibited significantly different nucleotide compositions of rRNA genes compared to the other three groups. The average AT contents of the two species in group C exceeded 76%, which were higher than the other three groups: 75.12%, 74.71% and 74.53% for groups A, B and D, respectively. In addition, the AT-skew and GC-skew of group C also showed intragroup specificity. The AT-skew of group C was -0.03, while all other 11 *Aparapotamon* species have an AT-skew of -0.01. These results demonstrated that in genes *16S rRNA* and *12S rRNA*, the two species in group C have a higher preference for base T than the species in the other three groups. In addition, the GC-skew of group C was -0.35 on average. In contrast, the values of nearly all other groups were higher than -0.4, indicating that the CG bias in Group C was not as significant as in other groups.

### Noncoding regions

On the basis of the current sequencing results, it is speculated that in the genus *Aparapotamon*, the putative control region is located between the *12S rRNA* and *tRNA-Met*, which contains two genes, *tRNA-Val* and *tRNA-Ile,* in the middle. Furthermore, the species with the longest putative region was *A. emineoforaminum*, with an estimated 3803 bp. The putative control region exhibited a high AT content of 80.93% on average in the genus *Aparapotamon*. In addition to the putative control region, three relatively long intergenic noncoding regions were observed in the arrangement of the genus *Aparapotamon*, which were located between genes *tRNA-Ser* and *ND1*, *16S rRNA* and *tRNA-Gln*, and *tRNA-Gln* and *12S rRNA* and were 245 bp, 235 bp, and 233 bp on average, respectively. Additionally, the arrangement of *16S rRNA, tRNA-Gln* and *12S rRNA* showed different patterns between the groups. The arrangement in group A was more compact, while the three genes in the other groups were relatively loosely arranged.

## Discussion

### Adaptive evolution characteristics of mitogenomes

After performing comparison analysis of the mitogenomes of all species in the genus *Aparapotamon*, we verified our conclusion of a four-group classification system in many aspects, and the adaptive evolutionary patterns of the genus *Aparapotamon* were discussed. At the same time, the results from comparative mitogenome analysis also corroborated the topological structures of the phylogenetic trees (Fig. 3, 4). For example, the relatively close relationship between groups B and D was confirmed not only by the phylogenetic trees but also by much related evidence found in their mitochondrial genomes, such as the similar base compositions, codon usage and similar mutations in tRNA genes.

During the multiple alignment of whole mitogenomes, the lengths of the noncoding region between different *Aparapotamon* species were unstable and varied to a large extent. In this region, there are a large number of simple sequence repeats (microsatellites), which can rapidly evolve due to slippage in replication [71, 72]. All species in group A exhibit significantly higher sequence identity of the noncoding regions, the sequence composition patterns of which could not be found in other groups (Fig. 5B). Meanwhile, specific arrangement patterns of the *tRNA-Ile* gene specific to group A were found (Fig. 6B). The intergenic regions between *tRNA-Ile* and *tRNA-Met* in group A were much shorter than those in the other groups. The same conditions also occurred in the arrangements of *16S rRNA, tRNA-Gln* and *12S rRNA*, which were more compact in group A than in the other groups. Due to the above characteristics, it can be speculated that the adaptive evolution of species in group A has left great imprints on this region.

In the analysis of the PCGs, the *ATP8* and *ND6* genes showed great diversity among all PCGs and evidence of having undergone positive selection. In the *ND6* gene, codon loss at position 416 was observed in all six species in group A, which can be considered a common characteristic preserved during the adaptive evolution of group A (Fig. 7). In the calculation of K2P distances, the interspecific genetic distances of *ND6* and *ATP8* were the longest, which reflected their relatively high rates of genetic variation during evolution in the genus *Aparapotamon* (Fig. 8B). For these two genes, the Ka/Ks values between species in group A and other species were all > 1, while the values between species in other groups were < 1. Considering the divergence time estimate obtained in a subsequent analysis, the divergence time within group A was the latest. This suggests that during the speciation of group A, for these two genes that experienced positive selection, some selective advantages emerged and were fixed in the population as a result of environmental adaptation [36]. According to the special geographical distribution patterns of the genus *Aparapotamon*, the altitudinal difference between different groups is obvious. Group C is distributed at high altitudes of 2400–2900 m, while group A, as the group at the lowest altitude, is distributed 500–1900 m above sea level. Therefore, species in the *Aparapotamon* genus are good materials for conducting research on altitude adaptation. Studies have reported the potential relationships between high-altitude adaptation and these two genes in other species, such as *ND6* in domestic horses [73] and *ATP8* in Tibetan loaches [74]. For freshwater crabs, our group first found that the *ND6* and *ATP8* genes may be related to adaptive evolution in response to altitude.

During the comparison of tRNA genes, it was found that *tRNA-Cys* has the maximum site number with intergroup differences, followed by *tRNA-Asn* and *tRNA-Ser (S1).* Combining the translation roles of tRNA, it can be speculated that these active tRNA genes may also be related to adaptive evolution in response to the environment within the genus *Aparapotamon*. *tRNA-Trp* and *tRNA-Thr* have only 1 site with intergroup differences and thus are relatively conserved during evolution. For the *16S rRNA* and *12S rRNA* genes, some characteristics specific to group C were observed, including a significantly higher AT content and CG bias. According to the results obtained here, species in group C branched first and were distributed at the highest altitudes. Therefore, during the process of species diversification and spread to lower-altitude and higher-latitude regions, adaptive evolution in response to some environmental changes appears to have left imprints on these two genes.

### Spatiotemporal biogeography

The geographical isolation caused by a series of geographical events, such as the uplift of the Qinghai–Tibet Plateau, the movement of the Himalayan plate and the uplift of the Hengduan Mountains [20, 75], affected the spread, genetic communication and species diversification of the genus *Aparapotamon*. The distribution pattern of the genus *Aparapotamon* is consistent with China's terrain ladders, with an altitude trend of high in the west and low in the east [76]. Overall, the genus gradually spread to areas of low altitude and high latitude along the Yangtze River, from China’s first terrain tier to the second terrain tier (Fig. 1).

Studies have shown that the Oligocene was the main period of Qinghai–Tibet Plateau formation, when geological movement was active and the plateau was uplifting [77, 78]. It is also believed that the last collision between the Indian Plate and Eurasian Plate occurred around the Miocene (25–20 Ma), which caused the eastern plate of the Qinghai-Tibet Plateau to move to the southeast, forming a complex terrain and climate in this region, with gradually obvious height differences between mountain ridges and valleys [79]. These complex geological changes had substantial impacts on species dispersal and isolation [80, 81]. According to the divergence time estimated in this study (Fig. 6A), approximately 18.1 Ma (95% credibility interval = 17.1–19.09 Ma), the genus *Aparapotamon* differentiated from *Potamiscus motuoensis*. Thus, the initial divergence of the genus *Aparapotamon* was very likely related to the changes in mountain terrain and rivers in the middle part of the Hengduan Mountains, which resulted in geographical isolation from the original ancestral populations. Then, the rapid uplift of the Hengduan Mountain Range on the southeastern Qinghai–Tibet Plateau [75, 81] further accelerated the dispersal and differentiation of the genus *Aparapotamon*, forming four groups: A, B, C, and D. Group C branched first 16.67 Ma (95% credibility interval = 15.77–17.59 Ma) and lives at the highest altitude among the four groups, in the middle part of the Hengduan Mountains with latitudes between 28° and 30° N. Groups B and D are mainly distributed on the Yunnan-Guizhou Plateau at altitudes lower than those of group C. The estimated divergence time between group D and group B was 11.82 Ma (95% credibility interval = 11.01–12.64 Ma). The divergence time of group A was approximately 15.56 Ma (95% credibility interval = 14.65–16.45 Ma); this group occupies the largest region, from 25°-33° N, and the lowest altitudes.

According to the species divergence time within each group, the differentiation of species in group A mainly occurred in the Pleistocene (3 Ma-1 Ma). During this period, glacial and interglacial stages were constantly alternating and changing, and the Hengduan Mountains underwent a series of glacial movements [22, 82]. Therefore, a series of deep valleys in the mountains provided important refugia for species during the glacial period [23, 83]. Because of adaptation to the changing environment, the rates of intraspecific evolution and species diversification in group A greatly increased.

### Evolutionary characteristics of species in group A

Group A is the group inhabiting the lowest altitudes and with the largest distribution and latest within-group estimated divergence time. Species in group A are mainly distributed along China’s secondary terrain tier (Fig. 1). It can be speculated that some species in group A, such as *A. grahami*, have spread outwards from the complex terrain of the Hengduan Mountains due to the occurrence of certain geological events, such as the uplift of mountains [20]. According to the mitogenome analysis in this study, it is highly likely that the adaptive evolutionary characteristics of group A appeared due to low-altitude environmental changes. Without the topographic restrictions of the Hengduan Mountain Range, species in group A spread to low altitudes to China’s second terrain tier and rapidly dispersed along the Yangtze River to the high-latitude regions, with a wider distribution and richer species diversity. This result suggests that the intercommunication of water systems may also contribute to the spread of freshwater crabs and thus influence their distribution, rather than them being absolutely dependent on geographical isolation.

The diversity of species and the fast evolutionary rate within group A have been confirmed in this study, including by gene arrangement patterns, Ka/Ks values and codon loss in the *ND6* gene. According to the Fauna Sinica (1999), large *A. grahami* specimens were collected from different locations, the G1 morphologies of which were slightly different [3]. Unfortunately, next-generation sequencing technology was not available at the time; otherwise, the active adaptive evolutionary mechanisms could be further studied by sequencing these precious specimens. As the only *Aparapotamon* species distributed in the central plains area in Henan Province, *A. gracilipedum* inhabits the lowest altitudes and shows the latest estimated divergence time in the genus. During the annotation of the obtained mitogenome sequences of *A. gracilipedum* (MZ350905), which was not sequenced by our laboratory, some new characteristics were found. First, the typical 7 bp overlaps between *ATP8* and *ATP6* and between *ND4* and *ND4L* could not be found in this sequence. A 7 bp duplication occurred in these two locations instead, and these two mutations could not be found in the other two *A. gracilipedum* mitogenome sequences. Due to the specific roles of these units in ATPase and NADH dehydrogenase assembly, mutations occurring in them may interfere with the respiratory chain and thus affect the function of mitochondria. The true reason for this cannot be known, i.e., whether there were real mutations in this individual sample or problems during sequencing and assembly. In addition, in this sequence, two cases of codon loss were observed, which made the PCG length of this sequence the shortest. In addition to the shared codon loss in *ND6* of group A, a codon loss event was also observed in *ND2*. Due to these features, we resequenced two additional *A. gracilipedum* samples. Then, we found that in *A. gracilipedum* (OP526650), the end of the *COX2* gene changed from TAA to AAA, which was followed by a single T that functioned as the new termination codon. This resulted in the addition of one more amino acid, lysine, at the end. All the evidence indicated that this species is in an active evolutionary state with the potential for complex subspecies differentiation.

From the geographical distribution, it can be speculated that after *A. grahami* spread to Henan, geographical isolation occurred due to the barriers created by various mountain ranges, which resulted in the speciation of *A. gracilipedum.* Here, this hypothesis was confirmed only by the phylogenetic tree constructed by using the three genes. Due to the relatively close relationship between these two specimens and the limited number of parsimony-informative sites in the three-gene phylogenetic trees, there may be uncertainty about this result. However, due to the lack of a complete mitogenome sequence of *A. grahami* distributed in the central plain areas, whether there is a direct evolutionary relationship between the two has not been confirmed.

## Conclusions

In this study, on the basis of analyses of the geographical distributions, morphology, phylogenetic relationships and mitogenomes of all 13 species in the genus *Aparapotamon*, a new pattern of species classification scheme within *Aparapotamon* was detected, in which the species were divided into four groups. Imprints from adaptive evolution were discovered in the mitochondrial genomes of group A. After some group A species dispersed from the Hengduan Mountain Range, new evolutionary characteristics emerged in their mitochondrial genomes. These evolutionary characteristics helped them adapt to the low-altitude environment of the second tier of China's terrain, after which they spread to high latitudes along the upper reaches of the Yangtze River, ultimately showing faster evolutionary rates, higher species diversity and the widest distribution.

The molecular characteristics of the mitochondrial genome involved in species diversification and adaptive evolution of the genus are discussed for the first time, which will help further the understanding of evolutionary mechanisms in freshwater crabs in China.

## Supplementary Information

**Additional file 1.**

**Additional file 2.**

## Data Availability

The mitogenomes sequencing data has been submitted on NCBI with accession numbers: *A. huizeense* (OP355466), *A. binchuanense* (OP355467), *A. grahami* (OM293968), *A. gracilipedum* (ON000286), *A. gracilipedum* (OP526650). The sequencing data used of gene *COX1*, *16S rRNA* and *28S rRNA* have also been submitted to NCBI and received the accession numbers (See Supplementary Table 2, Additional file 1).
