# Supplementary material for: Adaptive evolution characteristics of mitochondrial genomes in genus Aparapotamon (Brachyura, Potamidae) of freshwater crabs
Source: BMC Genomics. 2023 Apr 12;24:193. doi: 10.1186/s12864-023-09290-9 (PMC10091551; doi:10.1186/s12864-023-09290-9)
Supplement: Supplementary file 1 — Additional file 1. [file 12864_2023_9290_MOESM1_ESM.docx]

Supplementary Tables

**Supplementary Table S1.** Information of collected specimens of the genus *Aparapotamon* in China.

**Supplementary Table S2.** The sample information of genus Aparapotamon and GenBank accession numbers used for constructing three-gene phylogenetic trees (including outgroups).

**Supplementary Table S3.** Primer used in PCR process.

**Supplementary Table S4.** The Brachyura species used for constructing the 13 PCGs phylogenetic tree.

**Supplementary Table S5. The Kimura 2-parameter distance calculated based on 13 PCGs, including within-group mean distance and genetic distance between different groups.** There was no within-group mean distance in group D, as only one species, *A. emineoforaminum,* was classified into group D.

**Table S1. Information of collected specimens of the genus *Aparapotamon* in China.**

| Species name | Collection location | Latitude, longitude | Number |
| --- | --- | --- | --- |
| *Aparapotamon tholosum* | Lijiang, Yunnan | 100.821896°, 26.741154° | 9 |
| *Aparapotamon protinum* | Lijiang, Yunnan | 100.988074°, 26.998496° | 5 |
| *Aparapotamon gracilipedum* | Nanyang, Henan | 112.01778°, 33.465381° | 4 |
| *Aparapotamon huiliense* | Lufeng, Yunnan | 101.239872°, 26.742871° | 93 |
|  | Panzhihua, Sichuan | 102.118434°, 26.899979° | 29 |
|  | Panzhihua, Sichuan | 101.548959°, 26.597374° | 8 |
|  | Lufeng, Yunnan | 102.297717°, 25.215111° | 27 |
|  | Chuxiong, Yunnan | 101.993381°, 25.875432° | 22 |
|  | Chuxiong, Yunnan | 101.672239°, 26.060218° | 16 |
| *Aparapotamon grahami* | Zunyi, Guizhou | 106.956511°, 27.691995° | 13 |
|  | Hezhang, Guizhou | 104.689462°, 27.148311° | 36 |
|  | Bijie, Guizhou | 105.750731°, 26.678341° | 5 |
|  | Panzhihua, Sichuan | 101.644606°, 26.608875° | 21 |
|  | Zunyi, Guizhou | 106.9°, 27.7° | 1 |
|  | Dali, Yunnan | 100.82°, 24.42° | 11 |
|  | Yuxi, Yunnan | 102.75°, 24.09° | 6 |
|  | Wanzhou, Chongqing | 108.421542°, 30.788356° | 8 |
|  | Zunyi, Guizhou | 106.930077°, 27.691838° | 8 |
| *Aparapotamon binchuanense* | Dali Bai, Yunnan | 100.581328°, 25.826924° | 7 |
| *Aparapotamon huizeense* | Qujing, Yunnan | 103.27°, 26.41° | 8 |

**Table S2. The sample information of genus Aparapotamon and GenBank accession numbers used for constructing three-gene phylogenetic trees (including outgroups).** The number in this study corresponds to the number behind the *Aparapotamon* species name of the three-gene phylogenetic tree. The newly sequenced samples are highlighted in black bold.

| Species | Collection locality | Group | GenBank accession numbers | | | | | Number in this study | |
| --- | --- | --- | --- | --- | --- | --- | --- | --- | --- |
|  |  |  | *16S rRNA* | *COI* | | *28S rRNA* | |  | |
| ***A. grahami*** | **Zunyi, Guizhou** | **A** | **MZ489456** | | **MZ489481** | | **MZ492908** | | **GZ101** |
| ***A. grahami*** | **Zunyi, Guizhou** | **A** | **MZ489457** | | **MZ489482** | | **MZ492909** | | **GZ102** |
| ***A. grahami*** | **Hezhang County, Guizhou** | **A** | **MZ489458** | | **MZ489483** | | **MZ492910** | | **GZ103** |
| ***A. grahami*** | **Hezhang County, Guizhou** | **A** | **OM893788** | | **OM936016** | | **-** | | **GZ104** |
| ***A. grahami*** | **Bijie, Guizhou** | **A** | **MZ489459** | | **MZ489484** | | **MZ492911** | | **GZ105** |
| ***A. grahami*** | **Panzhihua, Sichuan** | **A** | **OM893789** | | **OM936017** | |  | | **SC101** |
| ***A. grahami*** | **Panzhihua, Sichuan** | **A** | **MZ489460** | | **MZ489485** | | **MZ492912** | | **SC102** |
| ***A. grahami*** | **Dali, Yunnan** | **A** | **OM893790** | | **-** | | **-** | | **YN102** |
| ***A. grahami*** | **Dali, Yunnan** | **A** | **OM893791** | | **-** | | **-** | | **YN103** |
| ***A. grahami*** | **Dali, Yunnan** | **A** | **OM893792** | | **-** | | **-** | | **YN104** |
| ***A. grahami*** | **Yuxi, Yunnan** | **A** | **MZ489464** | | **MZ489488** | | **MZ492916** | | **YN101** |
| ***A. grahami*** | **Zunyi, Guizhou** | **A** | **MZ489462** | | **-** | | **MZ492914** | | **GZ108** |
| ***A. grahami*** | **Chongqing** | **A** | **MZ489465** | | **MZ489489** | | **MZ492917** | | **CQ101** |
| ***A. grahami*** | **Chongqing** | **A** | **MZ489466** | | **MZ489490** | | **MZ492918** | | **CQ102** |
| ***A. grahami*** | **Zunyi, Guizhou** | **A** | **OM893793** | | **OM936018** | | **OM893777** | | **GZ106** |
| ***A. grahami*** | **Zunyi, Guizhou** | **A** | **-** | | **OM936019** | | **OM893778** | | **GZ107** |
| *A. grahami* | Yanglin, Yunnan | A | MZ350906 | | MZ350906 | | MZ338263 | | - |
| *A. grahami* | Yunnan | A | AB428489 | | - | | - | | - |
| ***A. tholosum*** | **Lijiang, Yunnan** | **B** | **MZ489448** | | **MZ489473** | | **MZ492900** | | **YN201** |
| ***A. tholosum*** | **Lijiang, Yunnan** | **B** | **MZ489449** | | **MZ489474** | | **MZ492901** | | **YN202** |
| *A. tholosum* | Yongsheng, Yunnan | B | MZ350914 | | MZ350915 | | MZ338270 | | - |
| ***A. protinum*** | **Lijiang, Yunnan** | **B** | **MZ489445** | | **MZ489470** | | **MZ492897** | | **YN301** |
| ***A. protinum*** | **Lijiang, Yunnan** | **B** | **MZ489446** | | **MZ489471** | | **MZ492898** | | **YN302** |
| *A. protinum* | Yongsheng, Yunnan | B | MZ350911 | | MZ350912 | | MZ338268 | | - |
| ***A. huiliense*** | **Lufeng County, Yunnan** | **A** | **OM893800** | | **-** | | **-** | | **YN414** |
| ***A. huiliense*** | **Lufeng County, Yunnan** | **A** | **OM893794** | | **OM936020** | | **-** | | **YN401** |
| ***A. huiliense*** | **Lufeng County, Yunnan** | **A** | **OM893795** | | **OM936020** | | **-** | | **YN402** |
| ***A. huiliense*** | **Lijiang, Yunnan** | **A** | **MZ489447** | | **MZ489472** | | **MZ492899** | | **YN403** |
| ***A. huiliense*** | **Lijiang, Yunnan** | **A** | **MZ489450** | | **MZ489475** | | **MZ492902** | | **YN407** |
| ***A. huiliense*** | **Lijiang, Yunnan** | **A** | **MZ489451** | | **MZ489476** | | **MZ492903** | | **YN408** |
| ***A. huiliense*** | **Lijiang, Yunnan** | **A** | **MZ489452** | | **MZ489477** | | **MZ492904** | | **YN409** |
| ***A. huiliense*** | **Lijiang, Yunnan** | **A** | **MZ489453** | | **MZ489478** | | **MZ492905** | | **YN410** |
| ***A. huiliense*** | **Lijiang, Yunnan** | **A** | **MZ489444** | | **MZ489469** | | **MZ492896** | | **YN404** |
| ***A. huiliense*** | **Chuxiong, Yunnan** | **A** | **MZ489454** | | **MZ489479** | | **MZ492906** | | **YN405** |
| ***A. huiliense*** | **Chuxiong, Yunnan** | **A** | **MZ489455** | | **MZ489480** | | **MZ492907** | | **YN406** |
| ***A. huiliense*** | **Panzhihua, Sichuan** | **A** | **-** | | **OM936021** | | **OM893779** | | **SC401** |
| ***A. huiliense*** | **Panzhihua, Sichuan** | **A** | **-** | | **OM936022** | | **-** | | **SC402** |
| ***A. huiliense*** | **Shaotong, yunnan** | **A** | **MZ489461** | | **MZ489486** | | **MZ492913** | | **YN411** |
| ***A. huiliense*** | **Panzhihua, Sichuan** | **A** | **OM893796** | | **OM936023** | | **OM893780** | | **SC403** |
| ***A. huiliense*** | **Panzhihua, Sichuan** | **A** | **OM893797** | | **OM936024** | | **OM893781** | | **SC404** |
| ***A. huiliense*** | **Chuxiong, Yunnan** | **A** | **OM893798** | | **OM936025** | | **OM893782** | | **YN412** |
| ***A. huiliense*** | **Chuxiong, Yunnan** | **A** | **OM893799** | | **OM936026** | | **OM893783** | | **YN413** |
| *A. huiliense* | Huili, Sichuan | A | MZ350907 | | MZ350908 | | MZ338264 | | - |
| ***A. huizeense*** | **Qujing, Yunnan** | **A** | **OM893801** | | **OM936027** | | **OM893784** | | **YN501** |
| ***A. huizeense*** | **Qujing, Yunnan** | **A** | **OM893802** | | **OM936028** | | **OM893785** | | **YN502** |
| ***A. huizeense*** | **Qujing, Yunnan** | **A** | **OM893803** | | **OM936029** | | **OM893786** | | **YN503** |
| ***A. binchuanense*** | **Dali, Yunnan** | **A** | **OM893804** | | **OM936030** | | **-** | | **YN601** |
| ***A. binchuanense*** | **Dali, Yunnan** | **A** | **OM893805** | | **OM936031** | | **OM893787** | | **YN602** |
| ***A. gracilipedum*** | **Nanyang, Henan** | **A** | **MZ489463** | | **MZ489487** | | **MZ492915** | | **HN701** |
| *A. gracilipedum* | Luanchuan, Henan | A | MZ350905 | | MZ350905 | | MZ338262 | | - |
| *A. molarum* | Lijiang, Yunnan | C | MZ350909 | | MZ350910 | | MZ338266 | | - |
| *A. emineoforaminum* | Mianning, Sichuan | D | MZ350904 | | MZ350905 | | MZ338261 | | - |
| *A. arcuatum* | Ninglang, Yunnan | B | MZ350903 | | MZ350904 | | MZ338260 | | - |
| *A. muliense* | Muli, Sichuan | B | MZ350910 | | MZ350911 | | MZ338267 | | - |
| *A. similium* | Yongsheng, Yunnan | A | MZ350912 | | MZ350913 | | MZ338269 | | - |
| *Tenuipotamon baishuiense* | Yunnan | - | MZ350952 | | MZ350952 | | MZ338292 | | - |
| *Tenuipotamon panxiense* | Yunnan | - | MZ350954 | | MZ350954 | | MZ338293 | | - |
| *Tenuipotamon yuxiense* | Yunnan | - | MN737140 | | MN737140 | | MZ338294 | | - |
| *Potamiscus motuoensis* | Yunnan | - | MN737138 | | MN737138 | | MZ338282 | | - |
| *Pararanguna semilinata* | Yunnan | - | MZ350936 | | MZ350936 | | MZ338279 | | - |
| *Parvuspotamon yuxiense* | Yunnan | - | MZ350939 | | MZ350939 | | MZ338280 | | - |
| *Potamiscus loshingensis* | Yunnan | - | MZ350941 | | MZ350941 | | MZ338281 | | - |
| *Geothelphusa dehaani* | - | - | AB187570 | | AB187570 | | AB503607 | | - |
| *Hainanpotamon orientale* | - | - | KT586288 | | KT586039 | | KT586428 | | - |
| *Neotiwaripotamon jianfengense* | - | - | KT586289 | | KT586040 | | KT586429 | | - |
| *Longpotamon denticulatum* | - | - | KT586100 | | KT585851 | | KT586323 | | - |

**Table S3. Primer used in PCR process.**

| Gene | Primer name | Primer sequence (5’-3’) | Expected length (bp) |
| --- | --- | --- | --- |
| *COX1* | FCOI | GTGGAAAGAGGTATTGGAACAGGATG | 1330 |
|  | RCOII | TCTATAAATGGGAGGCTGTGTCTTG |  |
| *16S rRNA* | 16S-1471 | CCTGTTTANCAAAAACAT | 550 |
|  | 16S-1472 | AGATAGAAACCAACCTGG |  |
| *28S rRNA* | F28S | CAGCCCTAAGCAGGTGGTAAACT | 1000 |
|  | R28S | CCACCATCTTTCGGGTCCCAACAT |  |

**Table S4. The Brachyura species used for constructing the 13 PCGs phylogenetic tree.** Species in *Aparapotamon* are highlighted in black bold. The newly sequenced species are marked by the star (*) after the name.

| Family | Species | Length（bp） | GenBank accession numbers |
| --- | --- | --- | --- |
| Portunidae | *Scylla olivacea* | 15723 | FJ827760 |
|  | *Scylla serrata* | 15721 | HM590866 |
|  | *Scylla paramamosain* | 15824 | JX457150 |
|  | *Scylla tranquebarica* | 15833 | FJ827759 |
|  | *Charybdis bimaculata* | 15714 | MG489891 |
|  | *Charybdis feriata* | 15660 | KF386147 |
|  | *Charybdis japonica* | 15738 | FJ460517 |
|  | *Charybdis natator* | 15664 | MF285241 |
|  | *Thalamita crenata* | 15787 | LK391945 |
|  | *Callinectes sapidus* | 16263 | AY363392 |
|  | *Portunus sanguinolentus* | 16024 | KT438509 |
|  | *Portunus pelagicus* | 16157 | KR153996 |
|  | *Portunus tritubercu Latus* | 16026 | AB093006 |
|  | *Monomia gladiator* | 15878 | MG770549 |
| Varunidae | *Helice latimera* | 16246 | KU589291 |
|  | *Helice tientsinensis* | 16212 | KR336555 |
|  | *Helicana wuana* | 16359 | KX344898 |
|  | *Cyclograpsus granulosus* | 16300 | LN624373 |
|  | *Eriocheir hepuensis* | 16335 | FJ455506 |
|  | *Eriocheir japonica* | 16352 | FJ455505 |
|  | *Eriocheir sinensis* | 16353 | KP126617 |
|  | *Varuna yui* | 15915 | MG756602 |
|  | *Hemigrapsus sanguineus* | 16275 | KX456205 |
| Bythograeidae | *Austinograea alayseae* | 15611 | KC851803 |
|  | *Austinograea rodriguezensis* | 15611 | JQ035658 |
|  | *Gandalfus puia* | 15548 | KR002727 |
|  | *Gandalfus yunohana* | 15567 | EU647222 |
|  | *Segonzacia mesatlantica* | 15521 | KY541839 |
| Sesarmidae | *Parasesarma tripectinis* | 15612 | KU343209 |
|  | *Clistocoeloma sinense* | 15706 | KU589292 |
|  | *Metopaulias depressus* | 15765 | KX118277 |
|  | *Sesarmops sinensis* | 15905 | KR336554 |
|  | *Sesarma neglectum* | 15920 | KX156954 |
| Potamidae | ***A. similium*** | **18043** | **MZ350912** |
|  | ***A. huiliense*** | **18186** | **MZ350907** |
|  | ***A. grahami**** | **17951** | **OM293968** |
|  | ***A. gracilipedum* A*** | **17988** | **ON000286** |
|  | ***A. gracilipedum* B*** | **17969** | **OP526650** |
|  | ***A. gracilipedum* C** | **16894** | **MZ350905** |
|  | ***A. binchuanense**** | **17995** | **OP355467** |
|  | ***A. huizeense**** | **17997** | **OP355466** |
|  | ***A. muliense*** | **19212** | **MZ350910** |
|  | ***A. arcuatum*** | **19128** | **MZ350903** |
|  | ***A. tholosum*** | **16605** | **MZ350914** |
|  | ***A. protinum*** | **17907** | **MZ350911** |
|  | ***A. inflomanum*** | **16287** | **MZ350908** |
|  | ***A. molarum*** | **19482** | **MZ350909** |
|  | ***A. emineoforaminum*** | **19432** | **MZ350904** |
|  | *Potamiscus motuoensis* | 17971 | KY285013 |
|  | *Geothelphusa dehaani* | 18197 | AB187570 |
|  | *Longpotamon xiushuiense* | 18460 | KU042041 |
|  | *Longpotamon yangtsekiense* | 17126 | KY785880 |
|  | *Sinopotamon yaanense* | 17885 | KY785879 |
|  | *Huananpotamon lichuanse* | 15380 | KX639824 |
|  | *Apotamonautes hainanensis bawanglingensis* | 17011 | MN737137 |
|  | *Candidiopotamon okinawense* | 17211 | MN737145 |
|  | *Longpotamon depressum* | 16537 | MW182411 |
|  | *Longpotamon exiguum* | 17324 | MW182410 |
|  | *Nanhaipotamon hongkongense* | 15318 | MW125541 |
|  | *Neilupotamon sinense* | 18894 | MN737143 |
|  | *Parapotamon spinescens* | 20227 | MN737144 |
|  | *Potamiscus yiwuensis* | 16307 | MN737136 |
|  | *Potamiscus yongshengensis* | 17821 | MN737142 |
|  | *Sinolapotamon patellifer* | 16547 | MK883709 |
|  | *Longpotamon parvum* | 19637 | MN737134 |
|  | *Longpotamon kenliense* | 18499 | MK584299 |
|  | *Chinapotamon maolanense* | 17130 | MT134100 |
|  | *Neilupotamon xinganense* | 16965 | MN117718 |
|  | *Bottapotamon lingchuanense* | 17612 | MN117717 |
|  | *Tenuilapotamon latilum kaiyangense* | 19294 | MW788029 |
| Raninidae | *Ranina ranina* | 15557 | AB752308 |
|  | *Lyreidus brevifrons* | 16112 | KM983394 |
|  | *Umalia orientalis* | 15466 | KM365084 |
| Homolidae | *Homologenus malayensis* | 15793 | KJ612407 |
|  | *Moloha majora* | 15903 | KT182069 |
| Majidae | *Maja crispata* | 16592 | KY650651 |
|  | *Maja squinado* | 16598 | KY650652 |
| Grapsidae | *Grapsus tenuicrustatus* | 15858 | KT878721 |
|  | *Pachygrapsus crassipes* | 15652 | KC878511 |
| Ocypodidae | *Ocypode cordimanus* | 15604 | KT896743 |
|  | *Ocypode ceratophthalmus* | 15564 | LN611669 |
| Xenograpsidae | *Xenograpsus testudinatus* | 15798 | EU727203 |
|  | *Xenograpsus ngatama* | 16106 | KY985236 |
| Macrophthalmidae | *Macrophthalmus japonicus* | 16170 | KU343211 |
| Mictyridae | *Mictyris longicarpus* | 15548 | LN611670 |
| Dotillidae | *Ilyoplax deschampsi* | 15460 | JF909979 |
| Gecarcinucidae | *Somanniathelphusa boyangensis* | 17032 | KU042042 |
| Mithracidae | *Damithrax spinosissimus* | 15817 | KM405516 |
| Matutidae | *Ashtoret lunaris* | 15807 | LK391941 |
| Xanthidae | *Atergatis floridus* | 16180 | MG792341 |
|  | *Atergatis integerrimus* | 15924 | MG786939 |
|  | *Leptodius sanguineus* | 15480 | KT896744 |
| Menippidae | *Myomenippe fornasinii* | 15658 | LK391943 |
| Geryonidae | *Chaceon granulatus* | 16135 | AB769383 |
| Eriphiidae | *Pseudocarcinus gigas* | 15515 | AY562127 |

**Table S5. The Kimura 2-parameter distance calculated based on 13 PCGs, including within-group mean distance and genetic distance between different groups.** There was no within-group mean distance in group D, as only one species, *A. emineoforaminum,* was classified into group D.

| Group | A | B | C | D | Within-group mean distance |
| --- | --- | --- | --- | --- | --- |
| A |  |  |  |  | 0.04 |
| B | 0.352 |  |  |  | 0.07 |
| C | 0.347 | 0.131 |  |  | 0.05 |
| D | 0.348 | 0.103 | 0.126 |  | - |
