# Supplementary material for: Adaptive evolution characteristics of mitochondrial genomes in genus Aparapotamon (Brachyura, Potamidae) of freshwater crabs
Source: BMC Genomics. 2023 Apr 12;24:193. doi: 10.1186/s12864-023-09290-9 (PMC10091551; doi:10.1186/s12864-023-09290-9)
Supplement: Supplementary file 2 — Additional file 2. [file 12864_2023_9290_MOESM2_ESM.pdf]

Supplementary Figures

Supplementary Fig. S1. Maximum Likelihood (ML) tree constructed by concatenated genes (*COX1+16S rRNA+28S rRNA*).

Supplementary Fig. S2. Bayesian Inference (BI) tree constructed by concatenated genes (*COX1+16S rRNA*+28S rRNA).

Supplementary Fig. S3. Maximum Likelihood (ML) tree constructed by the 13 PCGs.

Supplementary Fig. S4. Bayesian Inference (BI) tree constructed by the 13 PCGs.

Supplementary Fig. S5. The divergence time estimation based on the 13 PCGs.

Supplementary Fig. S6. The 95% credibility interval of estimated divergence time based on the 13 PCGs.

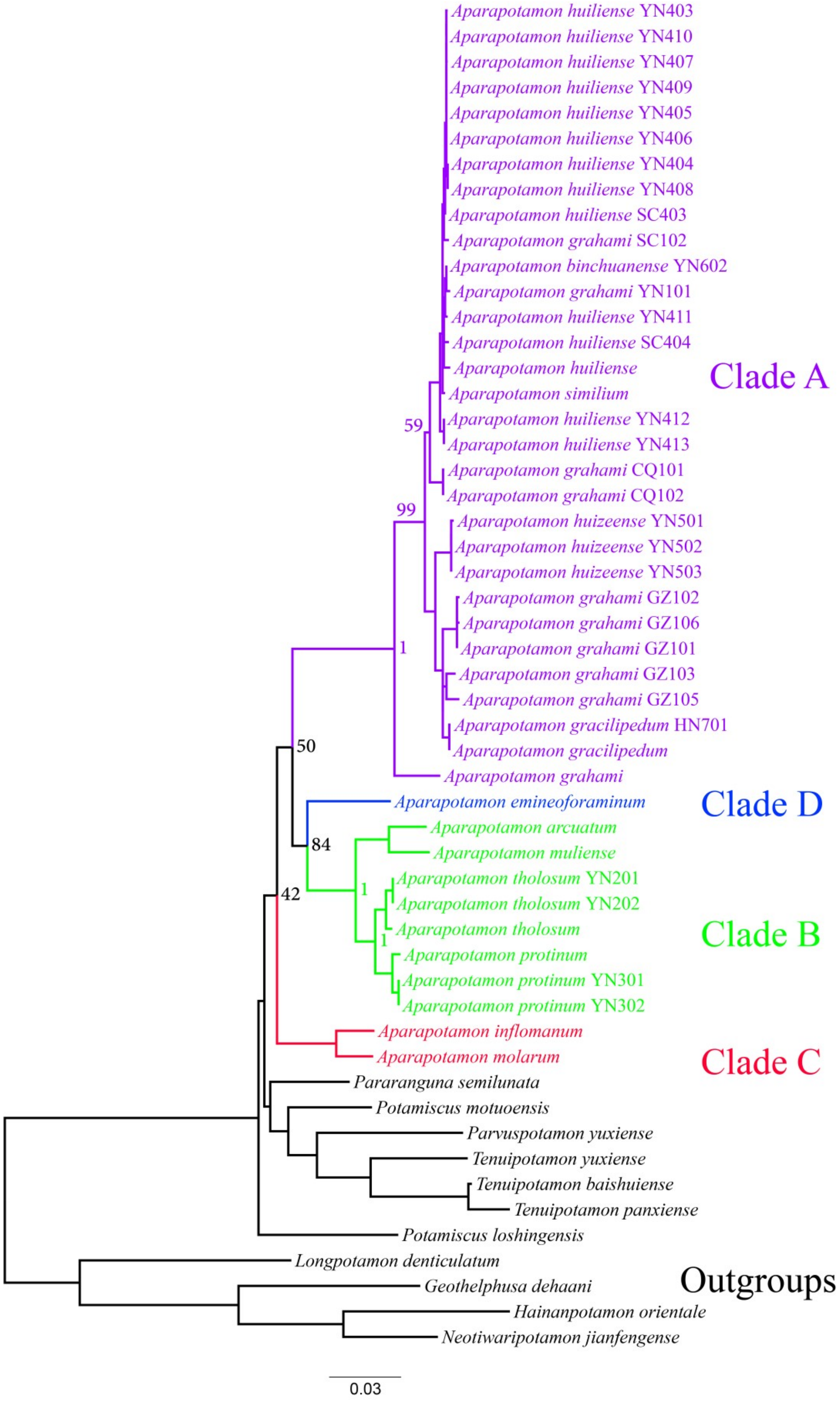

Fig. S1. Maximum Likelihood (ML) tree constructed by concatenated genes (*COX1+16S rRNA+28S rRNA*). The numbers at the internodes are maximum likelihood bootstrap proportions.

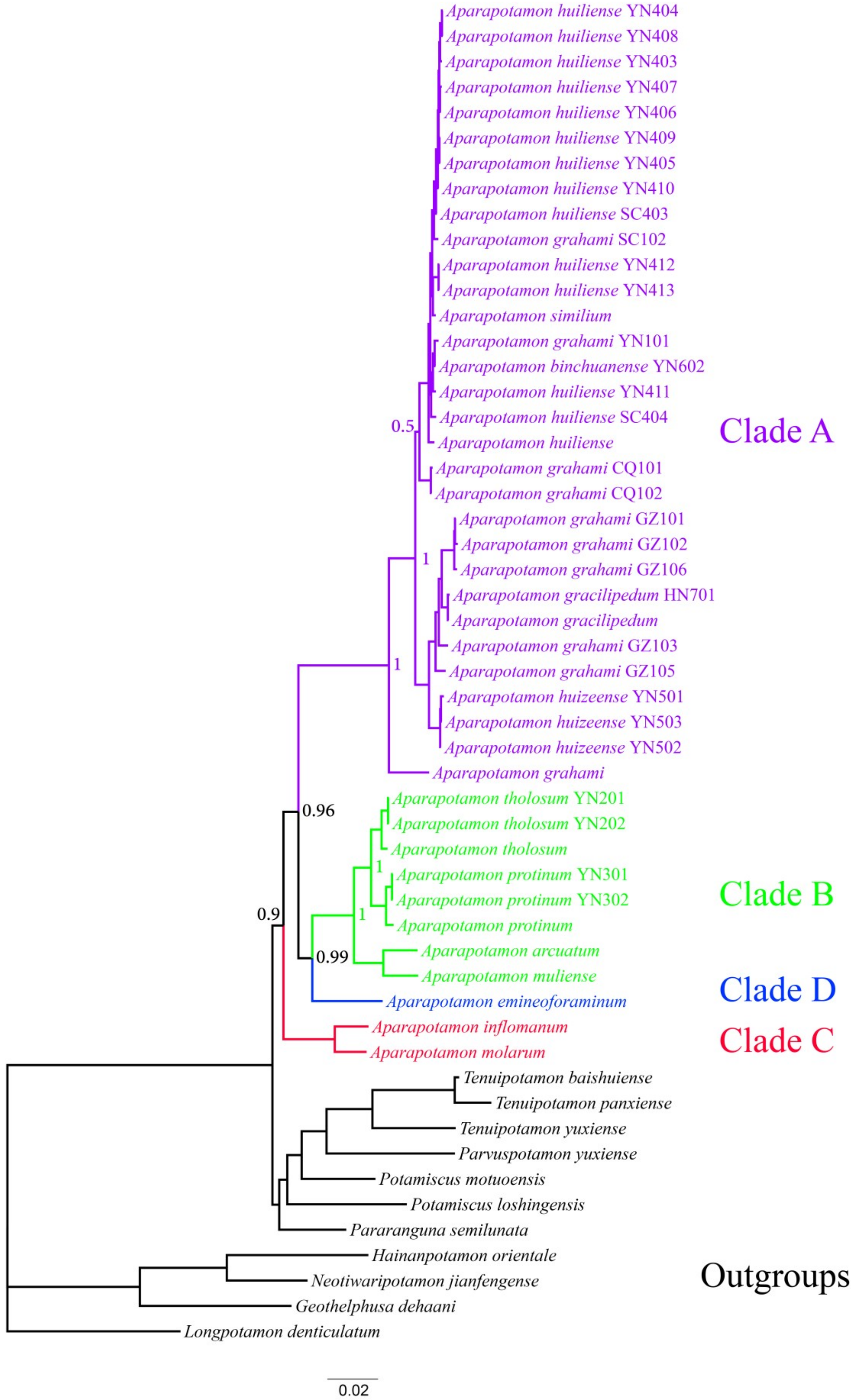

Fig. S2. Bayesian Inference (BI) tree constructed by concatenated genes (*COX1+16S rRNA+28S rRNA*). The numbers at the internodes are bayesian inference posterior proportions.

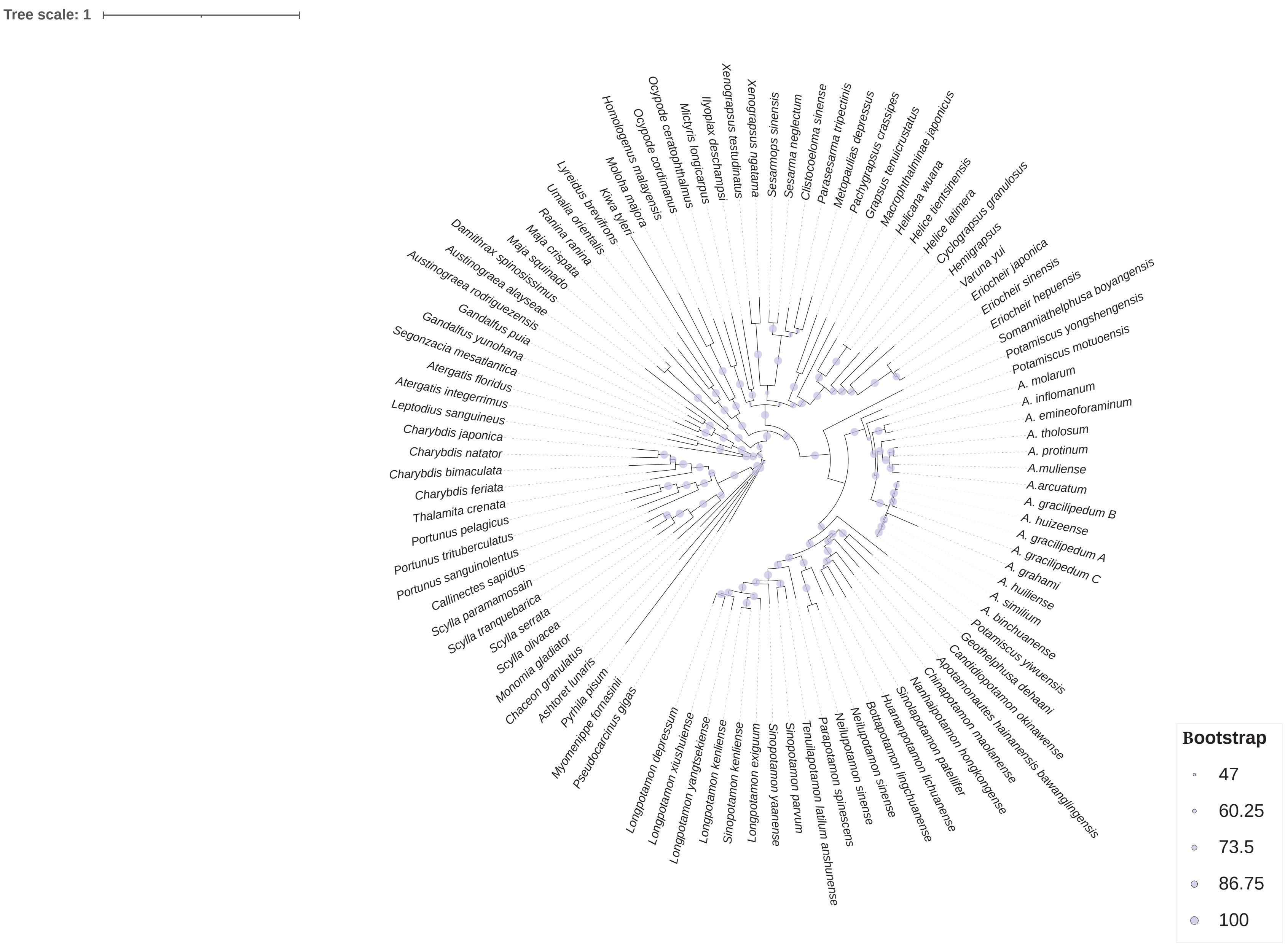

Fig. S3. Maximum Likelihood (ML) tree constructed by the 13 PCGs.

Fig. S4. Bayesian Inference (BI) tree constructed by the 13 PCGs.

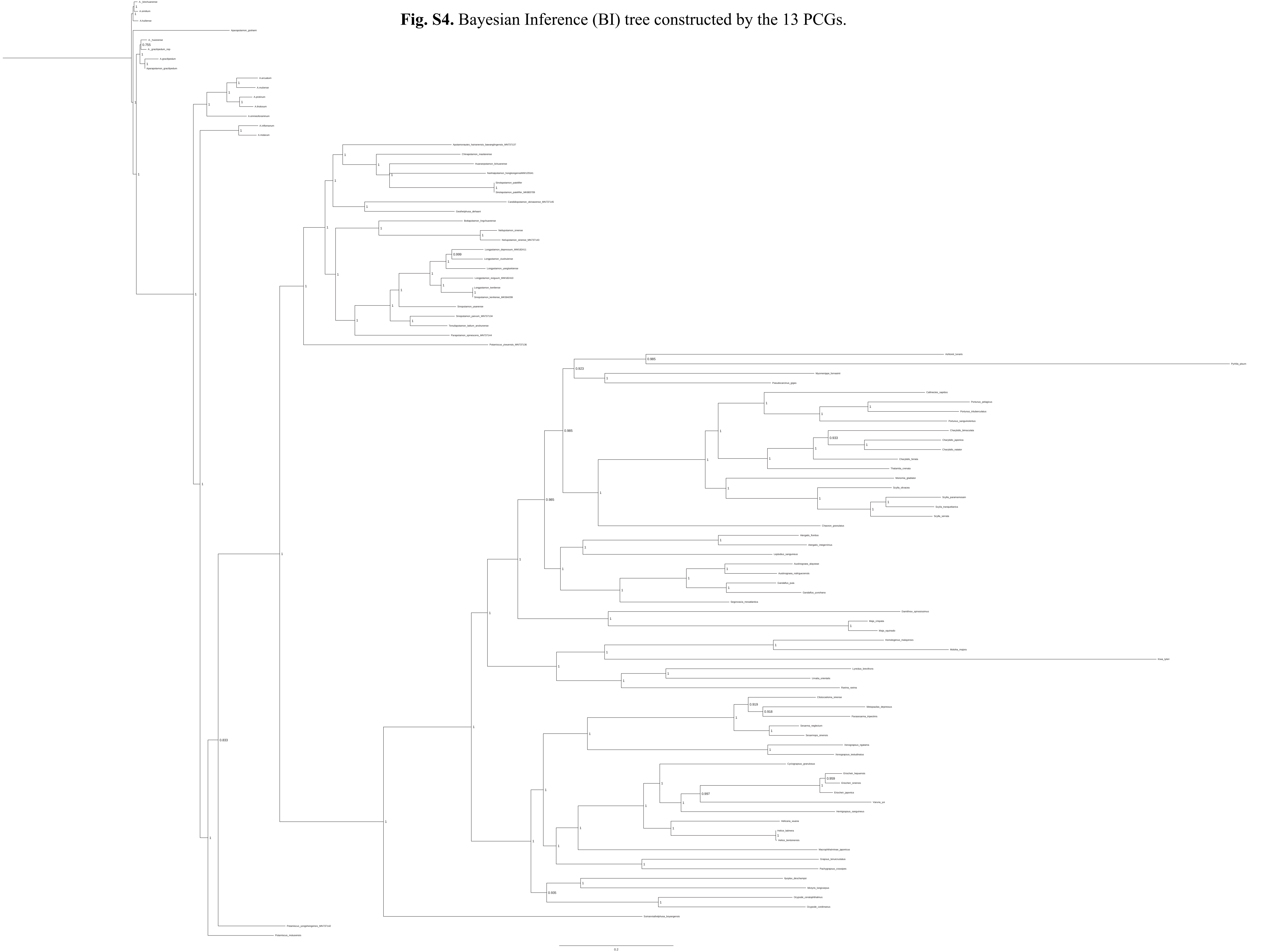

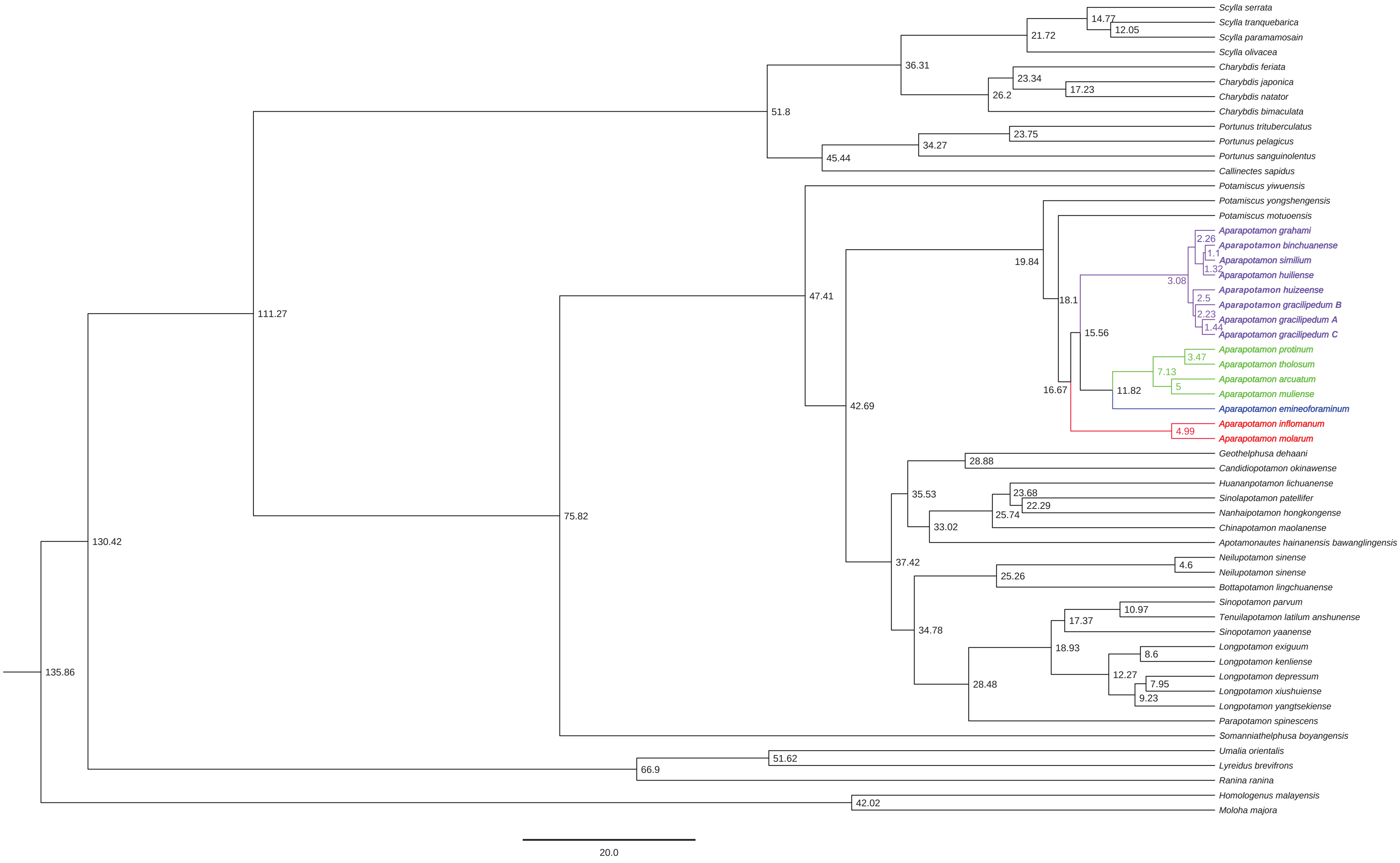

Fig. S5. The divergence time estimation based on the 13 PCGs.

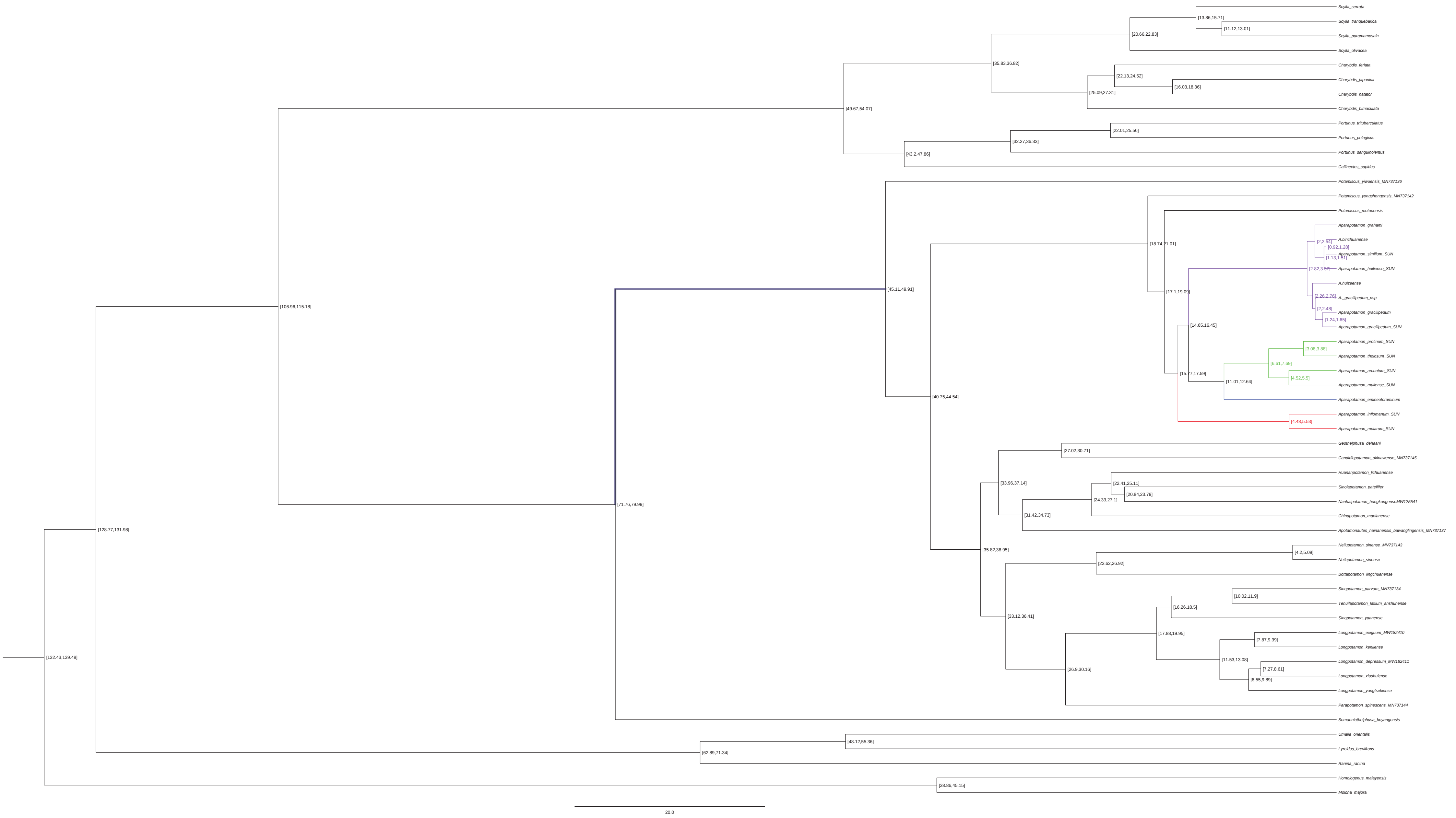

Fig. S6. The 95% credibility interval of estimated divergence time based on the 13 PCGs.
